# Building a Better Dynasore: The Dyngo Compounds Potently Inhibit Dynamin and Endocytosis

**DOI:** 10.1111/tra.12119

**Published:** 2013-10-09

**Authors:** Adam McCluskey, James A. Daniel, Gordana Hadzic, Ngoc Chau, Emma L. Clayton, Anna Mariana, Ainslie Whiting, Nick N. Gorgani, Jonathan Lloyd, Annie Quan, Lia Moshkanbaryans, Sai Krishnan, Swetha Perera, Megan Chircop, Lisa von Kleist, Andrew B. McGeachie, Mark T. Howes, Robert G. Parton, Michael Campbell, Jennette A. Sakoff, Xuefeng Wang, Jian‐Yuan Sun, Mark J. Robertson, Fiona M. Deane, Tam H. Nguyen, Frederic A. Meunier, Michael A. Cousin, Phillip J. Robinson

**Affiliations:** ^1^ Chemistry, Centre for Chemical Biology, School of Environmental and Life SciencesThe University of NewcastleCallaghanNSW2308Australia; ^2^ Cell Signalling UnitChildren's Medical Research Institute, The University of SydneyLocked Bag 23, WentworthvilleSydneyNSW2145Australia; ^3^ Centre for Integrative Physiology, Hugh Robson BuildingUniversity of EdinburghEdinburghEH8 9XDUK; ^4^ Department of Membrane Biochemistry, Institute of Chemistry and BiochemistryFreie Universität Berlin14195BerlinGermany; ^5^ Neuroscience Research AustraliaHospital RoadRandwickNSW2031Australia; ^6^ Institute for Molecular Bioscience and Centre for Microscopy and MicroanalysisUniversity of QueenslandBrisbaneQLD4072Australia; ^7^ Centre for Drug Candidate OptimisationMonash Institute of Pharmaceutical Sciences, Monash UniversityParkvilleVIC3052Australia; ^8^ Department of Medical OncologyCalvary Mater Newcastle HospitalEdith StreetWaratahNSW2298Australia; ^9^ Jian‐Yuan Sun Institute of BiophysicsChinese Academy of SciencesBeijingChina; ^10^ Queensland Brain InstituteThe University of QueenslandBrisbaneQLD4072Australia

**Keywords:** bulk endocytosis, drug discovery, dynamin, high‐throughput screening, small‐molecule inhibitors, synaptic vesicle endocytosis

## Abstract

**Dynamin GTPase activity increases when it oligomerizes either into helices in the presence of lipid templates or into rings in the presence of SH3 domain proteins. Dynasore is a dynamin inhibitor of moderate potency (IC_50_ ˜ 15 μM in vitro). We show that dynasore binds stoichiometrically to detergents used for in vitro drug screening, drastically reducing its potency (IC_50_ = 479 μM) and research tool utility. We synthesized a focused set of dihydroxyl and trihydroxyl dynasore analogs called the Dyngo™ compounds, five of which had improved potency, reduced detergent binding and reduced cytotoxicity, conferred by changes in the position and/or number of hydroxyl substituents. The Dyngo compound 4a was the most potent compound, exhibiting a 37‐fold improvement in potency over dynasore for liposome‐stimulated helical dynamin activity. In contrast, while dynasore about equally inhibited dynamin assembled in its helical or ring states, 4a and 6a exhibited >36‐fold reduced activity against rings, suggesting that they can discriminate between helical or ring oligomerization states. 4a and 6a inhibited dynamin‐dependent endocytosis of transferrin in multiple cell types (IC_50_ of 5.7 and 5.8 μM, respectively), at least sixfold more potently than dynasore, but had no effect on dynamin‐independent endocytosis of cholera toxin. 4a also reduced synaptic vesicle endocytosis and activity‐dependent bulk endocytosis in cultured neurons and synaptosomes. Overall, 4a and 6a are improved and versatile helical dynamin and endocytosis inhibitors in terms of potency, non‐specific binding and cytotoxicity. The data further suggest that the ring oligomerization state of dynamin is not required for clathrin‐mediated endocytosis**.

Dynamin is a large GTPase enzyme that severs membrane‐bound clathrin‐coated vesicles. Clathrin‐mediated endocytosis (CME) is involved in an array of vital cellular processes, including the internalization of activated receptors, sequestering growth factors, antigen presentation, cytokinesis, synaptic transmission and as an entry route for a variety of pathogens 1. There is now a new field of dynamin pharmacology with the development of multiple small‐molecule inhibitors specific for the dynamin family of GTPases as powerful new tools with which to study endocytosis. Small‐molecule dynamin inhibitors have attracted widespread attention and have been used to study endocytosis, other aspects of membrane dynamics and mitosis in a variety of cellular systems 1. Small‐molecule inhibitors offer many distinct advantages over traditional means of dynamin inhibition in cells by expression of dynamin GTPase mutants or by small interfering RNA (siRNA)‐mediated dynamin knockdown which cannot be used to study rapid cellular effects. Small‐molecule, cell‐permeable inhibitors can rapidly block endocytosis in minutes and their effects are typically reversible 3. Like all other research tools, they are subject to their own limitations, such as potentially poor cell permeability, cytotoxicity and risk of unknown off‐target actions. The field of targeted small‐molecule inhibitors of endocytosis has been recently expanded with the development of the Pitstop™ compounds, which are small‐molecule clathrin inhibitors 11.

The first reported dynamin inhibitors were long‐chain ammonium salts called MiTMAB™ compounds 12, followed by *dimeric tyrphostins* 13 and a series of room temperature ionic liquids (RTILs) 14. Two of the most potent inhibitors from the long‐chain ammonium salts, myristyl trimethyl ammonium bromide (MiTMAB) and octadecyltrimethyl ammonium bromide (OcTMAB), are potent and reversible inhibitors of endocytosis in neuronal and non‐neuronal cells, and selectively block dynamin's second function in cytokinesis 5. Dynamin is also inhibited by psychotropic drugs such as sertraline, a selective serotonin reuptake inhibitor 17. There now exists a small but expanding ‘palette’ of compounds available to rapidly and reversibly block dynamin by distinct mechanisms of action, thus operating at different stages in its cycle of GTPase activity. For example, MiTMAB, OcTMAB, RTILs and sertraline block dynamin recruitment to membranes, while the Dynole™ compounds and dynasore block dynamin after its recruitment 4. Structure–activity relationship (SAR) studies have afforded a range of inhibitors in the MiTMAB, RTILs and Bis‐tyrphostin (Bis‐T) series 10. The Dynole™ 4 the Iminodyn™ 21, Pthaladyn™ 22 and Pyrimidyn™ 23 series all have extensive SAR. Dynole 34‐2 (2‐cyano‐3‐(1‐(2‐(dimethylamino)ethyl)‐1*H*‐indol‐3‐yl)‐*N*‐octylacrylamide) is a 1.3 ± 0.3 μM dynamin inhibitor 4, three of the Iminodyn series and a second‐generation Dynole compound 2‐24 were the first nanomolar potent dynamin inhibitors 20.

No SAR has yet been reported for the dynamin inhibitor dynasore, which was discovered by a specific high‐throughput screen of a Chembridge library (DiversSet E) of 16 320 small molecules against grb2‐SH3 domain‐stimulated recombinant dynamin 3. Dynasore was originally reported to inhibit three forms of dynamin activity: (i) l‐phosphatidylserine (PS) liposome‐stimulated dynamin, which induces dynamin to form a helix around liposomes (note that we use the term ‘helix’ specifically to indicate the nanospring structure of dynamin, involving more than one turn of the ring); (ii) Grb2‐stimulated dynamin, the SH3 domains of which cross‐link dynamin tetramers into a potentially distinct conformational state involving single rings 24; and (iii) self‐assembly‐induced (SAI) activity, which is the basal activity stimulated up to 10‐fold by self‐assembly of dynamin into single rings, the formation of which is induced by high concentrations of dynamin in low salt buffers 26. However, the relative potency of dynasore in each of these systems was not investigated. The mechanism of dynamin inhibition by dynasore is not known, but the compound was reported not to affect GTP binding, dynamin self‐assembly, oligomerization or lipid binding 3. It blocked clathrin‐mediated endocytic functions that are well known to require dynamin, while it was without effect on clathrin‐ and dynamin‐independent endocytosis. Its ability to block endocytosis in a wide variety of cellular systems has been widely validated in the literature; however, it is not a potent in‐cell inhibitor.

Dynasore has undesirable non‐specific and specific binding properties in common with many other small‐molecule inhibitors. For example, it binds to serum proteins, causing it to lose dynamin inhibitory activity 27, limiting its use for many experimental designs. We show here that dynasore also exhibits stoichiometric binding to the trace level of detergents commonly used in biological assays. Concerned by this potential limitation, and with a background of previous development of functionally active dynamin and endocytosis inhibitors, we evaluated dynasore's SAR. By focusing on the role of the hydroxyl moieties 13, we developed a dynasore analog family with greatly reduced or no non‐specific *in vitro* binding and improved potency. By employing some elegantly simple medicinal chemistry strategies, the Dyngo™ series of compounds was generated. This includes a 37‐fold more potent dynamin and endocytosis inhibitor, Dyngo compound **4a**, and a wholly detergent‐resistant inhibitor, **6a**, both of which are more potent than the parent *in vitro* and in a diverse range of cellular endocytosis model systems. In preliminary reports, we noted that **4a** inhibits both dynamin I and II [IC_50_ for sheep brain dynamin I of 380 ± 0.05 nM (*n* = 5) and for recombinant rat dynamin II of 2.3 ± 0.2 μM (*n* = 3)], the internalization of botulinum toxin in nerve terminals 7, but not the clathrin‐independent carrier (CLIC) pathway 28. However, **4a** and related dynasore analogs have not been examined for CME or toxicity in non‐neuronal cells or for other modes of endocytosis. We now extensively characterize the development of **4a** and **6a**, from the Dyngo series of greatly improved dynasore analogs, as more versatile cell biology tools with reduced cytotoxicity.

## Results

### Dynasore quantitatively binds detergents

We developed a one‐step synthesis of dynasore (Figure S1A, Supporting Information) 27 and tested its ability to block the GTPase activity of dynamin I (purified from sheep brain). Under our standard PS liposome‐stimulated dynamin I assay conditions, we found that dynasore was effectively not an inhibitor of helical dynamin I GTPase activity (Figure S1B) with an IC_50_ of 479 μM (Figure S1C) and had no effect on dynamin II (up to 1.5 mM, not shown). This was much higher than the previously estimated IC_50_ for dynasore of ˜15 μM, although this value was obtained using grb2‐stimulated dynamin 3. To confirm that our relatively high IC_50_ was not a specific property of our in‐house‐synthesized dynasore, we verified this result using dynasore obtained commercially or from the original stock from the Kirchhausen laboratory (Figure S1B). Helical dynamin can also be stimulated with microtubules and ring dynamin by grb2‐SH3 domains or by self‐assembly; however, we found that dynasore did not block any of these activities (Figure S1C). Dynasore has also been previously found to be an inhibitor of CME 3, which we confirmed using an automated quantitative assay of transferrin‐A594 (Tfn‐A594) uptake in U2OS cells (Figure S1D), observing an IC_50_ of 34.7 μM. This verified the efficacy of dynasore in cells in our hands.

We noted that no detergents were used in the original dynasore study 3. Detergents are routinely used in assays to reduce non‐specific effects 29. Our standard GTPase assay contained 0.06% Tween‐80 (458 μM) and 20 nM dynamin I, suggesting that this may explain the discrepancy between our results and the original report of dynasore's efficacy 3. After redesigning our assay to accommodate no detergent (noting that such conditions greatly compromise the sensitivity and dynamic range of the *in vitro* assay), the IC_50_ for dynasore with PS‐stimulated helical dynamin I dramatically improved to 12.4 ± 1.5 μM (*n* = 5, Table 1). We also noted that increasing the dynamin concentration in the assay to 40 nM, the IC_50_ similarly increased to 73.6 ± 16 μM, indicating that IC_50_ values are dependent upon the dynamin concentration in the assay (as expected) and enzyme concentrations should be clearly noted in assay descriptions. The inhibition of full‐length helical dynamin II (recombinant protein expressed in Sf21 cells) was also restored when detergent was removed from the assay (IC_50_ = 18.1 ± 0.2 μM, *n* = 2). These findings suggested that dynasore binds to detergent with a stoichiometry of 1:1, because 0.06% Tween‐80 represents 458 μM detergent. This unfavorable property is reminiscent of a report that dynasore binds to serum proteins and similarly loses activity 27. Molecular modeling of dynasore bound to Tween‐80 suggested that the catechol moiety (i.e. the dihydroxybenzaldehyde) may be the major contributor to this interaction. As non‐specific binding greatly decreases the utility of any drug, we aimed to develop an improved dynamin inhibitor, using dynasore as a lead compound, by reducing detergent binding and improving potency.

**Table 1:** Dyngo compound **4a** inhibits dynamin I and CME

Name	Structure	Formula weight	DynI IC_50_ (μM) with T‐80	DynI IC_50_ (μM) without T‐80	CME IC_50_ (μM)
Library 1					
Dynasore	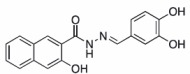	C_18_H_14_N_2_O_4,_ 322.31	479 ± 49 (*n* = 3)	12.4 ± 1.5 (*n* = 5)	34.7 ± 5.1 (*n* = 9)
**4a**	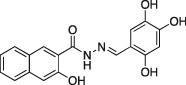	C_18_H_14_N_2_O_5,_ 338.31	2.7 ± 0.7 (*n* = 3)	0.38 ± 0.05 (*n* = 5)	5.7 ± 1.0 (*n* = 7)
**6a**	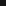	C_18_H_14_N_2_O_4,_ 322.31	5.5 ± 0.2 (*n* = 3)	3.2 ± 0.3 (*n* = 3)	5.8 ± 0.8 (*n* = 5)
**1a**	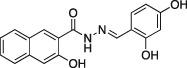	C_18_H_14_N_2_O_4,_ 322.31	37.4 ±0.9 (*n* = 3)	4.4 ± 1.0 (*n* = 3)	Not active (*n* = 3)
**5a**	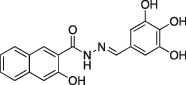	C_18_H_14_N_2_O_5,_ 338.31	102 ±14 (*n* = 2)	1.5 ± 0.04 (*n* = 2)	6.2 ± 2 (*n* = 2)
**2a**	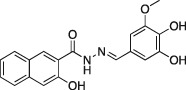	C_19_H_16_N_2_O_5,_ 352.34	Not active (*n* = 3)	3.3 ± 1.0 (*n* = 4)	9.6 ± 0.4 (*n* = 2)
**3a**	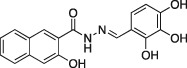	C_18_H_14_N_2_O_5,_ 338.31	Not active (*n* = 2)	1.5 ± 0.3 (*n* = 2)	9.8 ± 1.5 (*n* = 2)
**8a**	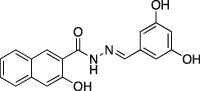	C_18_H_14_N_2_O_4,_ 322.31	Not active (*n* = 2)	47.0 ± 0.5 (*n* = 2)	179 ± 20 (*n* = 3)
Library 2					
**10a**	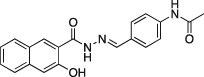	C_20_H_17_N_3_O_3,_ 347.37	Not active (*n* = 3)	39.5 ± 4.5 (*n* = 2)	Not active (*n* = 2)
**11a**	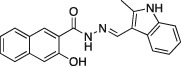	C_21_H_17_N_3_O_2,_ 343.38	58.9 ±1.4 (*n* = 2)	44.2 ±19.4 (*n* = 2)	63.4 ±4.4 (*n* = 2)
**12a**	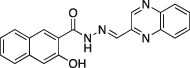	C_20_H_14_N_4_O_2,_ 342.35	>100 (*n* = 2)	24.6 ±4.1 (*n* = 2)	Not active (*n* = 2)
**13a**	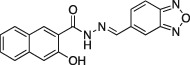	C_18_H_12_N_4_O_3,_ 332.31	30.6 ±5.0 (*n* = 6)	17.6 ± 3.5 (*n* = 2)	Not active (*n* = 3)
**14a**	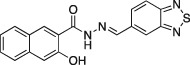	C_18_H_12_N_4_O_2_S_,_ 348.38	4.8 ±0.5 (*n* = 4)	11.6 ± 1.6 (*n* = 2)	Not active (*n* = 4)

A series of dynasore analogs (Dyngo compounds) were synthesized with substitutions in the (3,4‐dihydroxybenzylidene)‐hydrazide. Illustrated is the structure of each compound, its chemical formula, formula weight and IC_50_ for inhibition of native sheep brain dynamin I GTPase activity stimulated by PS liposomes, either in the presence or absence of Tween‐80 (T‐80) in GTPase assay. The last column shows the CME IC_50_ for inhibition of Tfn‐A594 uptake in U2OS cells after a 30‐min preincubation with each compound. All compounds were tested at multiple concentrations in 1% DMSO (in CME assay) and 3% (in GTPase assay) up to at least 1 mM concentration. Results are mean ± SEM, for *n* = 2–9 independent experiments.

### Building a better dynasore

The structure of dynasore (Figure S1A) is chemically similar to that of the Bis‐T series of dynamin modulators that we have previously reported 13. In that study, we found that the position and number of the hydroxyls around the phenyl ring contributed to their potency; thus, we used this as a template to assist in the development of a new series of compounds called the Dyngo compounds (Table 1). Each Dyngo compound was synthesized via a condensation reaction. Reaction of 3‐hydroxy‐2‐naphthoic acid hydrazide with a variety of mono‐, di‐ and tri‐hydroxybenzaldehydes afforded a highly focused Dyngo library 1 (**1a–8a**, Scheme S1).

Dynasore has two hydroxyls at C3′ and C4′ (on the phenyl ring, a 3′,4′‐catechol). When we initially screened the focused library on the dynamin I GTPase helix assays, the experiments were carried out in the presence of Tween‐80 (under which conditions dynasore returned an IC_50_ of 479 ± 49 μM). The results of this screen are summarized in Table 1. While three analogs, **2a**, **3a** and **8a** were inactive, all other Dyngo compounds exhibited increased inhibition compared to dynasore. In particular, the 2,3‐dihydroxy (**6a**) was about 90 times more potent than dynasore, with an IC_50_ of 5.5 μM, while the 2,4,5‐trihydroxyl analog (**4a**) was the most potent compound overall, with an IC_50_ of 2.7 μM (*n* = 3), making it 177 times more active than dynasore.

When the SAR for library 1 was re‐examined by GTPase assay in the absence of Tween‐80, markedly different results were obtained (Table 1). Most, but not all, Dyngo compounds exhibited up to 1000‐fold enhanced dynamin inhibition under the new assay conditions. Notably, the greatest detergent sensitivity was observed with analogs comprising at least one –OH moiety at either C3′ or C4′, namely dynasore (C3′ and C4′), **5a** (C3′ and C4′), **2a (C3 and C4)**, **3a** (C3′ and C4′) and **8a** (C3′). Thus, the number and position of the –OH moieties clearly influenced not only dynamin inhibition but also detergent sensitivity. The compounds with the lowest detergent sensitivity (defined as <10‐fold potency change on removal of Tween) were **4a**, **6a** and **1a**, each of which possessed a C2′‐OH. Both **2a** and **4a** contain a C4′‐OH moiety noted to be a major contributor to Tween sensitivity. **2a** lacks the free C5′‐OH and is 400‐fold Tween sensitive, whereas **4a** retains the C5′‐OH and is only sevenfold Tween sensitive. This suggests that the removal of the C4′‐OH or the inclusion of a C5′‐OH moiety would significantly reduce Tween sensitivity. **6a** has a C2′‐OH and no C4′‐OH, and is the most dynamin‐active, detergent‐insensitive analog (Table 1). Further examination of the molecular basis of detergent sensitivity led us to develop a second library of Dyngo compounds (**10a–14a**, Table 1, Scheme S1, Library 2), which confirmed the importance of –OH involvement in dynamin inhibition and detergent binding. These are discussed in detail in Supporting Information—Dyngo Library 2.

**Figure 1:**
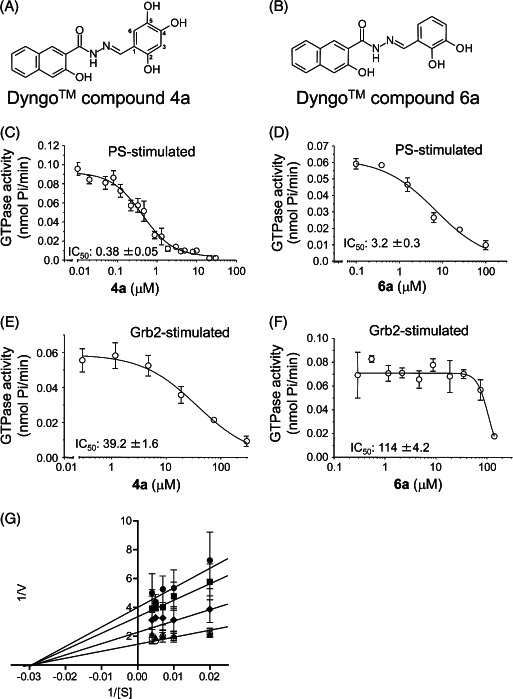
**Dyngo compounds 4a and 6a are potent inhibitors of** **PS**‐**stimulated dynamin helices.** A and B) Compound structures. C–F) Dose–response curves for inhibition of dynamin I GTPase activity by **4a** and **6a**. Helical dynamin I activity was stimulated by PS liposomes in (C) and (D) or ring dynamin I was stimulated by grb2 in (E) and (F). All data were obtained in the absence of Tween‐80. IC_50_ values are shown in μM (see also Tables 1 and 2). G) Non‐competitive kinetics of **4a** with respect to GTP. The data depict **4a** concentration‐dependent changes in a double‐reciprocal plot between substrate (GTP at 50–250 μM) and reaction velocity. The data correspond to **4a** concentrations at 6 (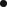), 5 (
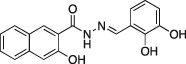
), 2.5 (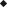), 1 (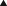) and 0.5 (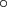) μM. Error bars represent the mean ± SEM of three independent experiments each conducted in triplicate.

In summary, we highlight analogs **4a** (Figure 1A) as the most potent and **6a** (Figure 1B) as being largely free of detergent effects. In the absence of detergent, **4a** exhibited a 37‐fold improved potency to 380 nM (Figure 1C), making it only the third submicromolar dynamin inhibitor yet reported, in addition to members of the Iminodyn and Dynole‐2 series 4. To optimally compare DynI and II we prepared full‐length recombinant versions of both enzymes in Sf21 insect cells and compared their IC_50_ values (Table 2). **4a** was 2.1‐fold selective for DynI versus DynII inhibition in the absence of Tween, and 3.1‐fold selective in its presence. **6a** was the second most potent inhibitor in our study and was equipotent in the presence and absence (Figure 1D and Table 1) of Tween‐80, highlighting its total lack of detergent binding.

**Table 2:** Relative IC_50_ values for inhibition of DynI and DynII by **4a**

**4a**	DynI (brain)	DynI (rec)	DynII (rec)	DynI selectivity ratio
Absence of Tween	0.38 ± 0.05 (5)	1.1 ± 0.2 (8)	2.3 ± 0.2 (4)	2.1
Presence of Tween	4.9 ± 0.9 (5)	30.0 ± 8.2 (2)	92.3 ± 10.9 (5)	3.1

DynI [from brain or recombinant (rec) protein from Sf21 cells] was used at 20 nM and DynII (recombinant protein from Sf21 cells) at 50 nM in the GTPase assay. The selectivity ratio refers to the DynI (rec) IC_50_ divided by that of DynII (rec). Data are mean ± SEM for the number of independent experiments shown in brackets.

### Differential inhibition of dynamin oligomerized in the helical or ring states

We next explored the capacity of the most potent analogs, **4a** and **6a**, to inhibit dynamin in different conformational states. It is known that both PS and microtubules support dynamin assembly into a helical shape (helical dynamin) along a structural template (tubulated lipid or rigid microtubules). PS uses the lipid‐binding properties of dynamin to assemble into a helix, while microtubules act as a scaffold or template for dynamin helix assembly 31. The helical oligomerization state of dynamin has been associated with CME in cells and so the capacity of Dyngo compounds to inhibit either helical or ring dynamin may be of potential biological importance in a cellular context. Dyngo series **4a** inhibited PS‐stimulated helical dynamin and microtubule‐stimulated dynamin with similar potency (IC_50_ of 2.7 versus 3.3 μM, respectively, in the presence of Tween‐80). This shows that **4a** does not inhibit by interfering with dynamin's lipid‐binding properties, demonstrating that it does not compete at the PH domain of dynamin.

Without a template for helical assembly, dynamin's GTPase activity can be greatly stimulated by SAI activity into single rings. However, the maximum activity of self‐assembled ring dynamin is 5‐ to 10‐fold less than that of helical dynamin; therefore, higher dynamin I concentrations (50 nM) were required for increased assay sensitivity. The SH3 domains of proteins such as grb2 stimulate dynamin SAI activity 24 as does dilution of dynamin into low salt buffers at high concentrations 26. In both situations, dynamin assembles as rings rather than a helix 24. In the absence of Tween‐80, dynasore inhibited grb2‐stimulated dynamin I with an IC_50_ of 38.2 μM, which is only threefold less potent than PS‐stimulated inhibition (Table 3, noting that slightly different dynamin I concentrations were used). This is consistent with the original dynasore report 3. Strikingly, both **4a** and **6a** were far less active against grb2‐stimulated ring dynamin (Figure 1E,F), with **4a** being 100‐fold less active and **6a** 36‐fold less potent (Table 3). Dyngo compound **4a** did not disrupt the binding of dynamin I to the SH3 domain of grb2 (Figure S2). Similarly, **4a** did not affect dynamin I binding to amphiphysin‐SH3 or endophilin‐SH3 domains (Figure S2). At high protein concentrations dynamin self‐assembles and is activated, but SAI activity in the presence of Tween (at 500 nM dynamin concentrations) was not at all inhibited by **4a**.

**Table 3:** Differential potency of dynamin inhibitors against grb2‐stimulated ring dynamin I

Compound	PS‐stimulated Dyn I, IC_50_ (μM)	Grb2‐stimulated Dyn I, IC_50_ (μM)
Dynasore	12.4 ± 1.5	38.2 ± 9.7
**4a**	0.38 ± 0.05	39.2 ± 1.6
**6a**	3.2 ± 0.3	114 ± 4.2

Data for grb2‐stimulated dynamin GTPase activity was obtained using 50 nM dynamin I (from brain). Data from PS‐stimulated dynamin (helix) used 20 nM (from Table 1). Data are from *n* = 6 (**6a**) or *n* = 4 (all others) experiments, using complete IC_50_ curves that were from independent experiments. All assays were conducted without Tween‐80.

To explore the mechanism of **4a**‐mediated inhibition of dynamin I GTPase activity, we conducted Michaelis– Menten kinetic experiments with **4a** and varying concentrations of GTP. Lineweaver–Burke plots demonstrated that **4a** is a non‐competitive inhibitor of dynamin I (Figure 1G), consistent with previous observations made examining the kinetics on dynamin inhibition by dynasore 3.

To examine whether the Dyngo compounds might bind to other key CME proteins that might account for its inhibitory actions, we performed *in vitro* assays to examine whether dynasore or **4a** and **6a** inhibit clathrin or AP‐2 protein interactions with amphiphysin I, both of which are key mediators of the early stages of CME. For the clathrin assay, the binding of clathrin heavy chain to amphiphysin 1 was examined. None of the compounds inhibited this interaction up to 300 μM (Figure S3A). The AP‐2 assay examined the binding of the AP‐2 alpha ear to amphiphysin 1. **6a** and dynasore had no effect, whereas **4a** had an IC_50_ of 362 μM, almost 1000 times less potent than for dynamin I inhibition (Figure S3B). Therefore, these compounds do not have off‐target actions on these two protein–protein interactions.

Overall, our findings demonstrate that the Dyngo compounds do not target the GTPase‐binding site within the G domain, do not inhibit the binding of three different SH3 domains that bind different sites in dynamin's PRD, do not require the function of the PH domain and do not have off‐target actions on protein–protein interactions involving amphiphysin I. Yet, we reveal that **4a** and **6a** inhibit helically assembled dynamin at least 36‐fold more potently than ring dynamin, in marked contrast to dynasore, thus failing to inhibit the distinct conformational state of ring dynamin.

### **4a** inhibits cellular endocytosis

Prior to an extensive study of endocytosis in a variety of cellular systems, we asked whether the lead Dyngo compounds might be toxic to cells in culture (Figure S4). Dyngo compounds **4a**, **6a** and dynasore did not exhibit generalized cytotoxicity after 8‐h exposure to HeLa cells assayed by lactate dehydrogenase (LDH) activity (Figure S4A,B). Prolonged exposure to Dyngo compounds (20 h) did not affect cell membrane integrity, as determined by a trypan blue exclusion assay (Figure S4C–F), nor did they induce apoptotic cell death (analyzed by flow cytometry in the quantitation of sub‐G1 peak or by western blotting for cleaved PARP, data not shown). These effects were unchanged in the presence or absence of cell culture serum. The small decrease in cell number after 20‐h exposure may suggest minor effects on cell growth or division, consistent with what are known effects of dynamin siRNA treatment 33 and its role in the final stage of mitosis, cytokinesis 34. Longer cell exposure of 72 h to **4a** and **6a** did not have any effect on cell viability or proliferation in a variety of cell lines using the standard MTT assay; however, dynasore showed broad‐spectrum toxicity in all the cells under these conditions (Table S1). Overall, **4a** and **6a** do not adversely affect cell viability and were markedly improved over dynasore under conditions of prolonged cell exposure for HeLa cells.

Dynasore was originally reported to inhibit the GTPase activity of dynamin‐like protein 1 (Dlp‐1), which is involved in mitochondrial fission. This raises the possibility that dynasore and the Dyngo compounds may affect mitochondrial morphology and/or dynamics in cells. We investigated this by labeling mitochondria in live HeLa cells using Mitotracker Green FM and imaging the cells over time using confocal microscopy. These cells stably expressed an mCherry‐conjugated form of the nuclear histone protein H2B, providing contrast to the green mitochondria. Images from cells treated for 30 and 60 min with either 30 μM **4a**, 30 μM **6a** or 100 μM dynasore are shown in Figure S5. Dyngo series **4a** and dynasore did not mediate any changes in mitochondrial morphology (Figure S4C,D). **6a** appeared to cause mitochondrial fragmentation (Figure S5E). The lack of any **6a** toxicity suggests that this mitochondrial fragmentation had no effect on cell viability. When cells were incubated in the presence of the compounds for longer than 30 min, all three compounds caused a reduction in Mitotracker Green FM fluorescence intensity, but the mechanism of which is unclear.

**Figure 2:**
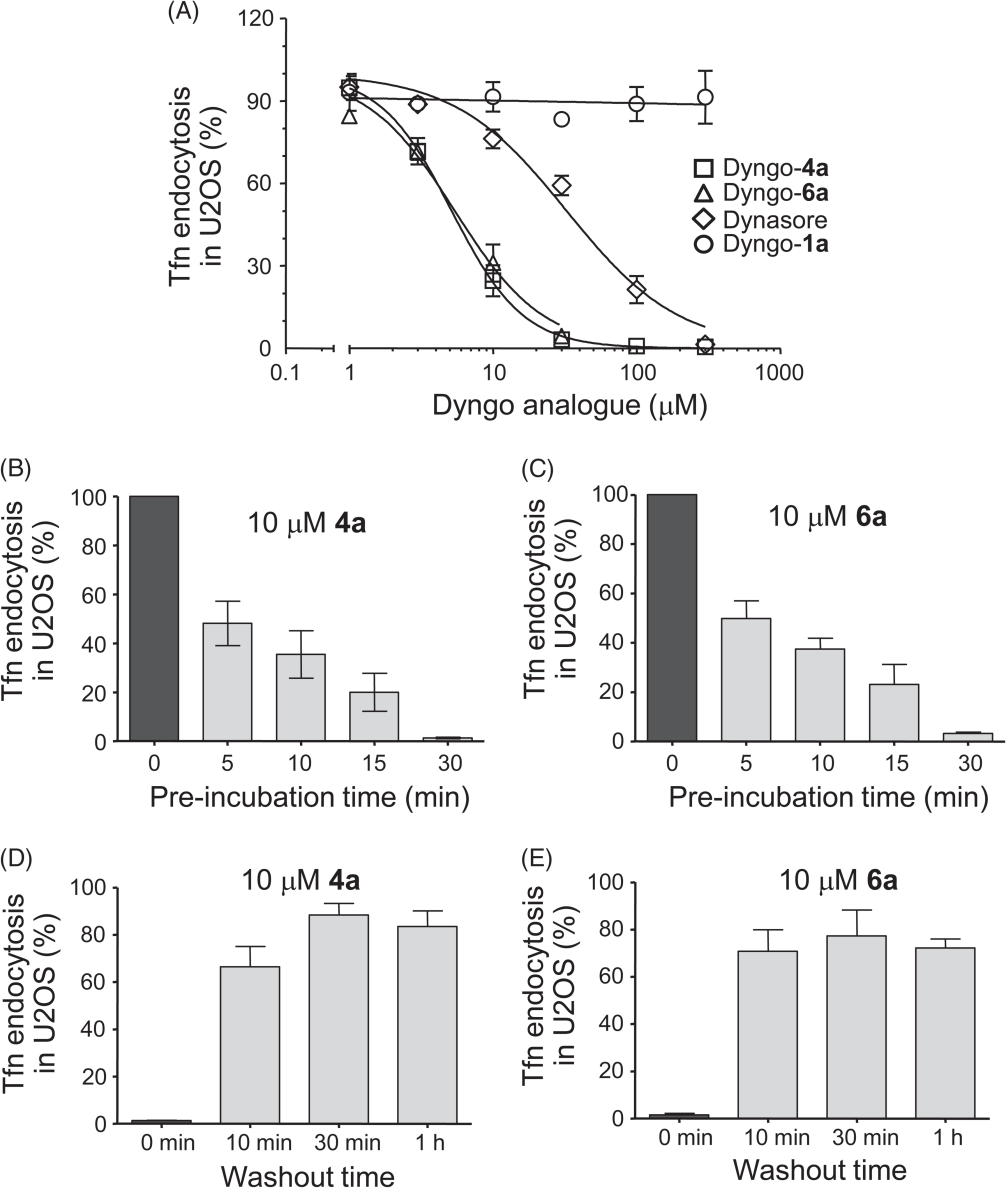
**Dyngo compounds are potent, reversible inhibitors of endocytosis in non‐neuronal cells.** A) The effect of three Dyngo analogs **4a**, **6a** and **1a** on endocytosis was compared with that of Dynasore (synthesized in‐house) by examining Tfn‐A594 uptake in U2OS cells. B and C) The time required for 10 μM **4a** (B) and **6a** (C) to inhibit non‐neuronal CME. Dyngo compounds were preincubated with cells before performing a Tfn uptake assay. D and E) Reversibility of endocytosis inhibition. The 10 μM **4a** (D) and **6a** (E) were incubated with cells for 30 min and then removed. Tfn uptake was then quantified at the indicated times after removal of the compound (washout time). Data are mean ± SEM of at least three independent experiments.

To determine whether the Dyngo compounds are widespread cell‐permeable and endocytosis inhibitors, CME of fluorescent Tfn was compared in an automated quantitative endocytosis assay in U2OS cells. The assay consisted of over 1200 cells under serum‐free conditions; after preincubation with each analog for 30 min the uptake of fluorescent Tfn was measured. We refer to this endocytosis as non‐neuronal CME to distinguish it from CME of synaptic vesicles in presynaptic terminals of neurons, which is referred to as synaptic vesicle endocytosis (SVE). In these cells, Tfn uptake is presumed to be mediated by dynamin II, because this is the only isoform of dynamin that they express, as determined by western blot (Figure S6). In the CME assay, **4a**, **6a** and **5a** were the most potent, being six times more potent than dynasore under the same conditions (Table 1). Dyngo compounds **2a** and **4a** were also active against non‐neuronal CME, whereas other analogs like **1a** (Figure 2A and Table 1) had little or no effect. There was no apparent correlation between *in vitro* and in‐cell activity, with potent dynamin‐active compounds like **1a** and **14a** having no effect on non‐neuronal CME (Table 1). This might be explained by low membrane permeability of these analogs, their cellular metabolism or rapid cellular efflux mechanisms. Dyngo compounds **4a** and **6a** potently inhibited Tfn endocytosis with IC_50_ values of 5.7 ± 1.0 μM and 5.8 ± 0.8 μM, respectively, approximating the activity of the most potent small‐molecule endocytosis inhibitors previously reported [*Dynole* compound 34‐2 4 and Iminodyn compound 22 21]. We also investigated this further in NIH3T3 cells across a range of **4a** and dynasore concentrations, confirming that these compounds selectively inhibit dynamin‐dependent endocytosis with much less effect on the uptake of cholera toxin (CT; Figure S7). This is consistent with our previous preliminary electron microscopy report in fibroblasts cells that **4a** does not inhibit the uptake of CT by CLICs, which is dynamin‐independent 28.

We next examined the exposure time of U2OS cells to the compound before an endocytic block was achieved, using **4a** or **6a** at 10 μM. Inhibition of non‐neuronal CME was observed with as little as 5‐min preincubation with **4a**, although 30 min was required for complete abolition of Tfn uptake (Figure 2B). The same trend was obtained with **6a** (Figure 2C). Therefore, 30‐min preincubation was used for most subsequent experiments. We then tested whether the endocytic block with **4a** and **6a** was reversible. Cells were incubated with 10 μM of the compound for 30 min, before its removal and assay of non‐neuronal CME by Tfn uptake. This ‘wash out’ allowed us to determine the length of time required for recovery of normal non‐neuronal CME. Ten minutes after the removal of **4a**, we observed a recovery to 70% of normal non‐neuronal CME levels (Figure 2D). Longer wash out times (up to 1 h) resulted in over 80% recovery. In the same experiment using **6a**, we found that non‐neuronal CME was restored to 70% of the normal level after 60 min of wash out (Figure 2E). Hence, the actions of **4a** and **6a** are rapid and reversible.

Dynasore is reported to lose non‐neuronal CME inhibition in cell culture after exposure to serum 27. Given that **4a** and **6a** exhibited reduced detergent binding, we examined whether serum binding was also reduced compared to dynasore. All three compounds bound strongly to human serum albumin (**4a**: 99.4% bound, **6a**: 99.7% and dynasore: 98.8% bound), suggesting that the *in vitro* non‐specific binding differences between them relate to Tween‐80 rather than to albumin. We next examined whether **4a** shows improved non‐specific binding by assaying non‐neuronal CME in the presence of serum or albumin protein. Dynasore lost virtually all CME inhibitory activity when used in the presence of 10% FBS or 1% BSA for 60 min (Table 4), extending a previous report 27. In contrast, **4a** and **6a** were still active in the presence of 5 or 10% FBS or 1% BSA but were at least 20‐fold less potent (Table 4). Reducing the FBS to 1% or the BSA to 0.1% increased the potency of all three compounds; however, there was still about 10‐fold less endocytosis inhibition compared to the absence of serum protein (Table 4). When taking their dynamin potency into consideration, the effects of Dyngo compounds and dynasore on endocytosis are reduced to similar extents by serum or albumin. Therefore, although Dyngo compounds remain more active than dynasore in the presence of serum or albumin, this may simply be owing to their improved potency.

**Table 4:** Effect of albumin on CME potency of the Dyngo compounds

Compound	1 h	10% FBS	5% FBS	1% FBS	1% BSA	0.1% BSA
Dynasore	22.2 ± 6.0	532	650	57.8	235	277
**4a**	6.3 ± 3.6	115 ± 7.5	102	23.7 ± 3.2	83 ± 12	44
**6a**	3.3 ± 0.6	108 ± 15	78 ± 7	16.5	281	46

Table shows IC_50_ values for inhibition of CME in U2OS cells after incubation of cells for 1 h in the presence or absence of FBS or BSA and the indicated compound. Data are mean (μM) and SEM or range of two to four independent experiments (1 h, *n* = 3; 1% FBS, *n* = 2; 10% FBS, *n* = 4).

### Dyngo compounds inhibit synaptic vesicle endocytosis

**Figure 3:**
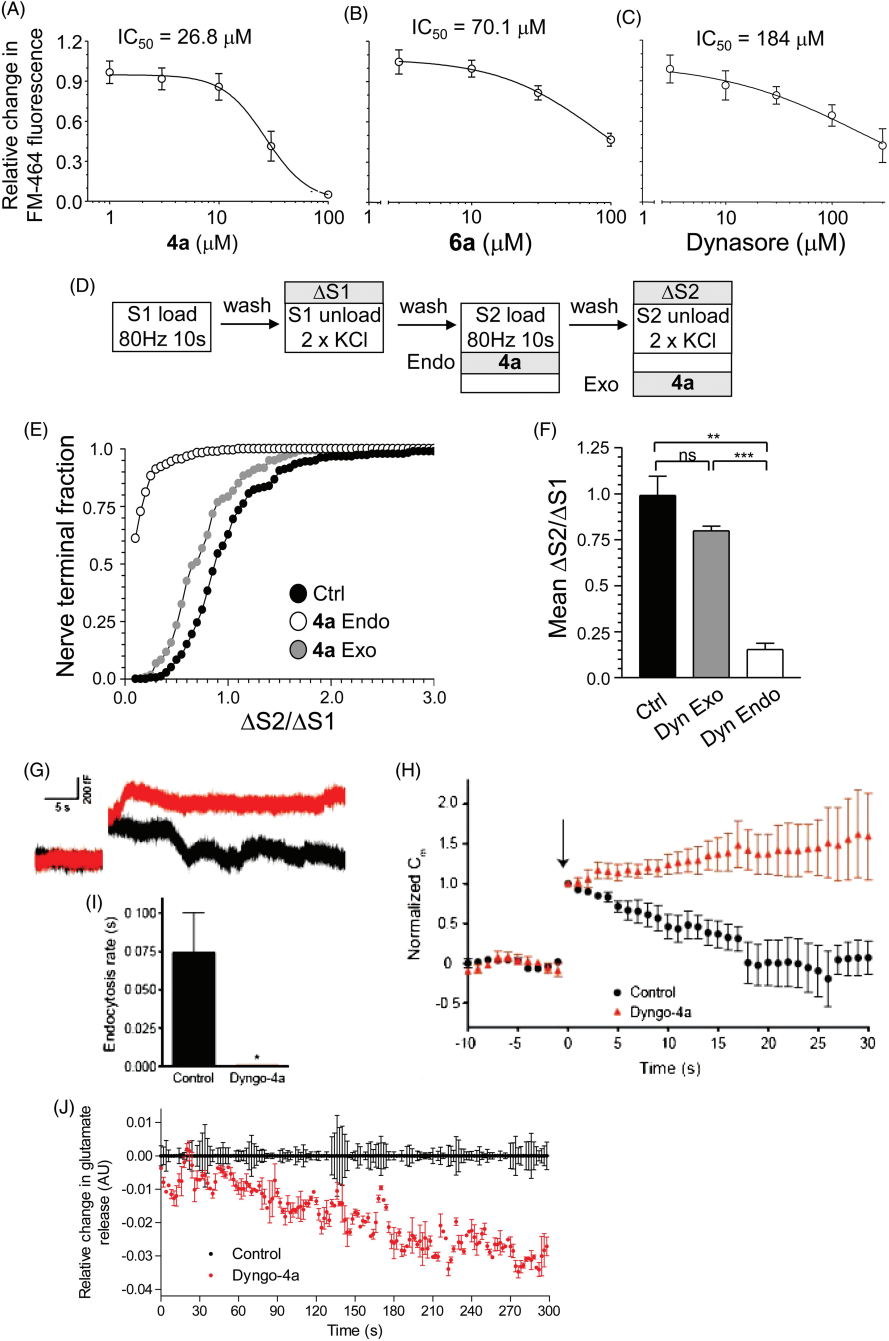
**Dyngo compound 4a inhibits** **SVE** **in synaptosomes and neurons.** A–C) SVE was examined by quantifying uptake of FM4‐64 in synaptosomes stimulated with 40 mM KCl. Dose–response curves and IC_50_ values are shown for **4a** (A), **6a** (B) and dynasore (C). D–F) SVE in cultured CGNs. D) To further examine SVE inhibition by **4a**, CGNs were loaded and unloaded with FM1‐43 using the protocol displayed. In both S1 and S2 load phases, dye was loaded into retrieving synaptic vesicles with 800 action potentials (80 Hz for 10 seconds). Unloading was stimulated by two sequential 30‐second stimuli using 50 mM KCl. The extent of SV turnover was estimated from the total amount of dye unloading at S1 (ΔS1) and S2 (ΔS2). Where indicated, cultures were preincubated with 30 μM **4a** for 15 min prior to and during either S2 loading (Endo) or unloading (Exo). E) Cumulative histograms display the ratio of ΔS2 to ΔS1 across a population of single synapses. Black circles show untreated control data (Ctrl). Dyngo compound **4a** was either applied during the loading phase, quantifying the effect of **4a** on SVE (open circles), or during the unloading phase, which quantifies the effect on exocytosis (gray circles). F) The mean ΔS2/ΔS1 response (±SEM) is displayed for control cultures (black bar, n = 270 nerve terminals) and for cultures where **4a** was present in either the S2 unload (gray bar, n = 163, exocytosis) or S2 load (open bar, n = 270, endocytosis). Dyngo compound **4a** had no significant effect on exocytosis, but significantly inhibited endocytosis, one‐way ANOVA, **p < 0.01, ***p < 0.001. G and H) The effect of **4a** on whole‐cell membrane capacitance was investigated at the Calyx of Held with 0.3 mM **4a** in the puffing pipette. G) A sample trace shows membrane capacitance on control (black) and **4a**‐treated samples (red). H) Collated data of normalized capacitance measurement from control and **4a**‐treated neurons (n = 6). Dyngo compound **4a**‐treated samples showed no inhibition in exocytosis but a dramatic reduction in endocytosis. I) Rate of membrane retrieval (paired t‐test, p = 0.048). All data except (E) and (G) are means ± SEM. J) Ca^2+^‐dependent exocytosis from synaptosomes was measured after 30‐min incubation in 1% DMSO (control) or 20 μM **4a**. To examine whether an activity‐dependent decrease in glutamate release was apparent with **4a** treatment, the average control values were subtracted from each data point, thus representing the control sample as a relative change in glutamate release of zero. Dyngo compound **4a** had no effect for the first 45–50 seconds, and subsequently caused a reduction in stimulated exocytosis that increased with stimulation time, suggesting activity‐dependent depletion in the pool of releasable synaptic vesicles in synaptosomes. Data are mean from two experiments ± range.

Much of the research on the involvement of dynamin in endocytosis has focused on SVE. SVE has many features in common with the non‐neuronal CME pathway, but is primarily mediated by dynamin I, whereas non‐neuronal CME of Tfn is mediated by dynamin II. We examined whether **4a** and **6a** inhibited SVE (Figure 3). A dose–response for inhibition of SVE was established by examining uptake of the styryl dye FM4‐64 in rat brain synaptosomes using a 96‐well microplate assay 35. Dyngo compound **4a** inhibited SVE with an IC_50_ of 26.8 μM (Figure 3A, *n* = 6) and **6a** with an IC_50_ of 70.1 μM (Figure 3B, *n* = 6). Dynasore was seven times less potent, with an IC_50_ of 184 μM under the same conditions (Figure 3C, *n* = 3). Inhibition of SVE by **4a** was further investigated in cultured cerebellar granule neurons (CGNs). SVE was measured by the activity‐dependent loading and unloading of the styryl dye FM1‐43 (Figure 3D, representative images are shown in Figure S8A–D). After incubation with 30 μM **4a** for 15 min, the loading of FM1‐43 was inhibited, suggesting a blockade in synaptic vesicle turnover, i.e. the number of synaptic vesicles undergoing exocytosis and then endocytosis (Figure 3E). This is consistent with previous reports that dynasore does not affect synaptic vesicle exocytosis but blocks SVE 2. To confirm that the block of SV turnover by **4a** was due to the arrest of SVE, its effect on FM1‐43 unloading (SV exocytosis) was determined. Dyngo compound **4a** did not have a significant acute effect on SV exocytosis, indicating that its predominant action was the specific inhibition of SVE (Figure 3F).

Dyngo series **4a** inhibition of SVE was investigated in neurons in situ within brain slices by studying depolarization‐induced changes in membrane capacitance at the Calyx of Held. Control recordings showed a typical rapid increase in membrane capacitance at the onset of stimulation, indicative of exocytosis of synaptic vesicles, followed by a decline in capacitance associated with SVE (Figure 3G,H, black traces). Dynasore (100 μM, 5‐min preincubation) has been previously shown to inhibit SVE in this experimental system 36, and we reproduced similar observations (Figure S9) using acute and direct application of 800 μM dynasore in a puffing pipette. We next examined the effects of **4a** at a lower concentration (using direct application of 300 μM in the puffing pipette) than was used for dynasore. These concentrations were selected to account for direct drug application rather than via preincubation. The decaying phase of the trace was abolished, suggesting that **4a** caused a complete blockade of SVE (Figure 3G,H, red traces). Treatment with **4a** did not acutely affect exocytosis. This data provides further evidence of specific inhibition of SVE by **4a**. The capacitance trace increased slightly over time after the initial capacitance jump of exocytosis with **4a** treatment. This may indicate further accumulation of fused vesicles at the plasma membrane, increasing the membrane capacitance in the absence of any compensatory SVE. Overall, this data shows that **4a** is a more potent CME/SVE inhibitor compared to dynasore in diverse neuronal and non‐neuronal cellular systems.

Exocytosis is known to rundown after prolonged stimulation, while endocytosis is blocked owing to a SV rundown 2. We investigated the effects of **4a** on glutamate release from synaptosomes during long repetitive stimulation (Figure 3J). Synaptosomes were pretreated with 20 μM **4a** and Ca^2+^‐dependent glutamate release was stimulated by 3 mM 4‐aminopyridine (4‐AP), which causes repetitive action potential like stimulation 38. Dyngo compound **4a**‐treated synaptosomes demonstrated a decrease in the release of glutamate only after 45 seconds of 4‐AP stimulation. As Dyngo compound **4a** does not inhibit exocytosis directly (Figure 3F), this suggests that inhibition of SV recycling produces an activity‐dependent rundown in synaptic transmission.

### Dyngo compound **4a** inhibits both modes of SVE

**Figure 4:**
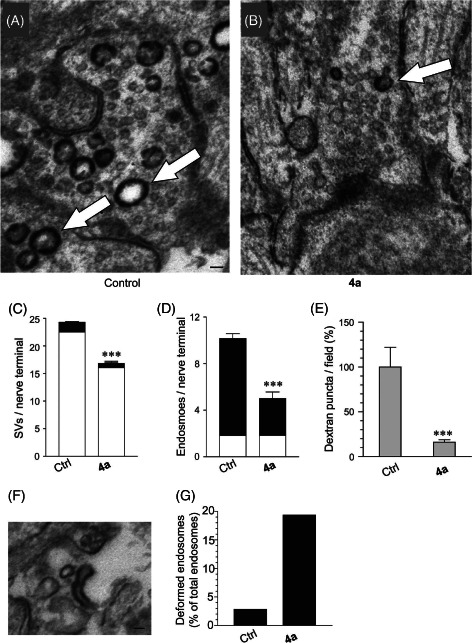
**Dyngo compound 4a inhibits both** **CME** **and** **ADBE.** Uptake of HRP in response to electrical stimulation (80 Hz for 10 seconds) was examined in CGNs. Neurons were preincubated with or without 30 μM **4a** for 15 min before and during stimulation. Panels show typical electron micrographs of CGNs after HRP labeling and fixation, either in the absence (A) or presence of **4a** (B). HRP‐labeled endosomes are indicated by arrows. Scale bar represents 100 nm. Panel (C) shows quantitation of the number of synaptic vesicles observed in control and **4a**‐treated samples. Synaptic vesicles were either unlabeled (white bar) or HRP labeled (black bar). **4a** treatment significantly reduced the number of unlabeled and labeled synaptic vesicles, demonstrating an inhibition of CME. Panel (D) shows quantification of labeled and unlabeled endosomes. **4a** inhibited the number of HRP‐labeled endosomes, demonstrating an inhibition of ADBE. E) **4a** (30 μM) inhibited uptake of fluorescent dextran in CGNs electrically stimulated at 80 Hz, further suggesting inhibition of ADBE. F and G) In electron micrographs, **4a** treatment also increased the appearance of deformed HRP‐labeled endosomes, such as that shown in (F). Scale bar represents 50 nm. Quantification of the number of deformed endosomes for each condition is shown in (G), presented as % of total endosome number. All data are means ± SEM, ***p < 0.001, Student's t‐test.

There are two distinct modes of SVE in neurons: neuronal CME and activity‐dependent bulk endocytosis (ADBE) 39. We investigated whether **4a** inhibited both modes in cultured CGNs by delivering high‐intensity electrical stimulation in the presence of horseradish peroxidase (HRP), which facilitates labeling of endocytosed structures with an electron‐dense substrate (Figure 4A,B). Pretreatment with 30 μM **4a** caused a significant reduction in the number of labeled synaptic vesicles (Figure 4C), indicating that neuronal CME was inhibited in these neurons. Unlabeled synaptic vesicles were also reduced, indicating a lack of replenishment of pools by neuronal CME (Figure 4C). The incomplete abolition of SVE by **4a** at a fixed 30 μM concentration is in line with our earlier observation that the IC_50_ for SVE inhibition is 26.8 μM. In addition, **4a** caused a decrease in the number of HRP‐labeled endosomes, indicative of a block in ADBE (Figure 4D). Using a more quantitative assay the reduction in ADBE by **4a** was confirmed in experiments showing that 30 μM of the compound reduced the uptake of large fluorescent dextrans (which are too large to accumulate in synaptic vesicles) in cultured CGNs (Figure 4E, representative images are shown in Figure S8E,F). Furthermore, a large number of the residual HRP‐labeled endosomes formed in the presence of **4a** display an elongated tubular structure, rather than a typical spherical appearance (Figure 4F,G). These malformations indicate that even when ADBE occurs, the formation of bulk endosomes shows some defects, as previously observed with 100 μM dynasore 40 and in nerve terminals of dynamin I knockout mice 41. The malformations are reminiscent of the shape of CLICs defined by electron microscopy in fibroblasts, and this endocytic pathway was previously shown to be unaffected by **4a** 28. We did not observe complete abolition of ADBE by 30 μM **4a** treatment (being a maximum of >80% block measured by dextran uptake). This might be explained by the fact that a concentration of **4a** close to the IC_50_ was used.

## Discussion

We report a library of dynasore analogs with greatly increased potency, significantly reduced non‐specific binding characteristics, which are not cytotoxic and have a markedly improved potency in cells. The best Dyngo series compound, **4a**, is shown to inhibit not only non‐neuronal CME but also SVE and ADBE at presynaptic nerve terminals. Surprisingly, the Dyngo compounds exhibit a marked preference for inhibition of dynamin in its helical conformation, while dynasore showed little preference. Helical dynamin is thought to be the form of dynamin responsible for facilitating endocytosis in cells. These compounds thus have broader utility and greater versatility for cell biologists. We first found that dynasore stoichiometrically binds to detergents routinely used for *in vitro* GTPase assays. Simple modifications of the substituents and substitution pattern of dynasore's catechol moiety produced a range of improved dynamin GTPase inhibitors, with **4a** and **6a** showing the optimum properties. Dyngo compounds **4a** and **6a** exhibit little to no (respectively) residual detergent binding. Unlike dynasore, **4a** and **6a** exhibit greater potency for inhibiting PS‐stimulated helical dynamin compared to grb2‐stimulated ring dynamin, demonstrating a preference for inhibition of the helical conformation, which has been directly observed at the neck of vesicles undergoing endocytosis in cells. Using the same assay conditions, **4a** is 37 times more potent than dynasore against dynamin I and is 6 times more potent in blocking dynamin‐dependent endocytosis, while not affecting dynamin‐independent endocytosis. Dyngo series **6a** shows intermediate *in vitro* potency, yet is equally potent to **4a** in non‐neuronal cells. Dyngo compound **6a** has the advantage of retaining no detectable non‐specific detergent interaction. We propose that **4a** and **6a** are improved, versatile, cell‐permeable dynamin I and II inhibitors. These findings demonstrate that simple substitutions within the dynasore pharmacophore have had profound effects on the specificity and cellular activity of the compound and highlight a small compound group with reduced non‐specific interactions.

In high‐throughput screening for enzyme modulators, detergents are routinely included in enzymatic assays because many small molecules will cause proteins to precipitate, leading to a very high rate (up to 95%) of false‐positive hits when a compound library is screened 43. Inclusion of detergent prevents small‐molecule‐induced aggregation of the target enzyme. However, dynasore was discovered by screening a library of 16 320 small molecules for inhibition of grb2‐stimulated dynamin activity 3. This screen was performed in the absence of any detergents, such as Tween‐80, which we use in our dynamin assay, or Triton X‐100 (which potently inhibits dynamin GTPase activity and cannot normally be used in dynamin assays *in vitro*). Had detergent been included, as is routine practice in recent years, dynasore would not likely have been discovered, owing to its unusual detergent‐binding properties, which render it essentially inactive. We also found that dynamin itself was rendered inactive by the inclusion of Triton X‐100, but not Tween, at very low concentrations down to 0.01%. These observations highlight the critical importance of the conditions used for any high‐throughput assay, in that the chosen conditions may act as a limiting factor in the type of molecules that will be detected as hits. Therefore, while conservative approaches, such as using detergent, are generally recommended, it should not be assumed that a high‐throughput assay is capable of detecting all important leads within a compound library.

Dyngo compounds **4a** and **6a** have a distinct activity profile from dynasore in their ability to distinguish between the activities of helical versus ring dynamin *in vitro*. Dynamin has at least two distinct oligomerization states: (i) rings, which form in the presence of proteins with SH3 domains or with F‐actin and is a widespread property within the larger dynamin superfamily, and (ii) helices, which form in the presence of lipid or microtubule templates and require the PH domain (which is not part of the sequence of non‐classical dynamins). It is not yet known which regions of dynamin are responsible for helical assembly, as opposed to single ring assembly, but is likely to involve the middle domain and/or bundle signaling element 46. Dynasore was initially discovered through a screen for compounds that inhibit grb‐stimulated ring dynamin and does not appear to discriminate ring or helix dynamin. The mechanism of dynamin inhibition by dynasore is not known, but the compound was reported not to affect GTP binding, dynamin self‐assembly, oligomerization or lipid binding. The authors suggested that it targets the GTPase domain 3. We have largely confirmed these studies for dynasore, but found that the dynamin inhibitory action of the family of Dyngo compounds is strongly dependent on the specific oligomerization state of dynamin. Compound **4a** was without effect on SAI activity stimulated by removing salt and was over 100‐fold weaker on grb2‐stimulated activity, both of which are mediated by ring formation. In contrast, it was about equally potent against dynamin stimulated by PS liposomes and microtubules, both of which facilitate helical assembly of dynamin. Thus, the mechanism of dynamin inhibition by **4a**/**6a** appears to be distinct from dynasore and involves preferential interference with the function of a helix versus dynamin oligomerization to the single ring state. This mechanism of action is novel for an enzyme inhibitor. A specific biological function for dynamin rings distinct from that of the helix has not yet been reported. However, the reduced activity of Dyngo compounds on dynamin rings, while retaining potent anti‐CME activity, indicates that ring function is not directly associated with any of the modes of CME investigated in our study. Dynamin rings are known to be induced by short F‐actin assemblies 51, raising the possibility of a role for dynamin ring oligomerization in actin dynamics.

Our discovery of improved dynamin inhibitors, with narrower activity profiles and that also block endocytosis, significantly adds to the growing palette of reported dynamin modulators developed by our group (MiTMAB, RTIL, Dynole, Iminodyn, Pthaladyn, Rhodadyn, Pyrimidyn and Dyngo compounds) and provides two new discovery tools for biomedical researchers. These dynamin inhibitors include compounds with distinct mechanisms of action at the PH (pleckstrin homology) domain (MiTMAB and RTILs), allosteric sites on the G domain (Dynole and Dyngo compounds), GTP binding (Pthaladyn compounds) or that have dual action at both GTP and lipid binding (Pyrimidyn compounds). We suggest that the most appropriate use of small‐molecule inhibitors in cell‐based studies is to employ more than one structurally distinct inhibitor 52, and this stringency can be increased by also using inhibitors that target different dynamin domains. This strategy increases the probability of revealing a dynamin‐dependent cellular process and reduces the possibility of unexpected off‐target drug actions. The use of dynamin inhibitors with different mechanisms of action in combination with each other provides investigators with a powerful toolkit for more accurate molecular dissection of the role of dynamin in multiple endocytic pathways and cytokinesis 1. For example, the combined use of **4a** or **6a** with Dynole compound 34‐2 should be more valuable in teasing apart the molecular steps of endocytosis, owing to their very distinct mechanisms of actions within the same clathrin‐mediated endocytic pathway.

## Materials and Methods

### Materials

PS, phenylmethylsulfonylfluoride (PMSF) and Tween‐80 were from Sigma‐Aldrich. GTP was from Roche Applied Science and leupeptin was from Bachem. Gel electrophoresis reagents, equipment and protein molecular weight markers were from Bio‐Rad. Collagenase was obtained from Roche. Paraformaldehyde (PFA) was from Merck Pty Ltd. Coverslips were from Lomb Scientific. Penicillin/streptomycin, phosphate‐buffered salts, fetal bovine serum (FBS), Dulbecco's minimal essential medium (DMEM), Alexa‐594‐conjugated Tfn (Tfn‐A594), DAPI, FM4‐64, Calcein blue‐AM and FM2‐10 were from Life Technologies. All other reagents were of analytical reagent grade or better.

Endogenous dynamin I (18 mg) was purified from sheep brain as described 10 and full‐length human dynamin I or rat dynamin II (both N‐terminally His‐6‐tagged) was recombinantly expressed from a pIEx‐6 vector in insect cells (Sf21) using polyethyleneimine (25 kDa) as the transfection reagent using a DNA:polyethyleneimine ratio of 1:5 for 48 h and purified by affinity purification on GST‐Amph2‐SH3‐sepharose 21. Recombinant dynamin I was used only for the data in Table 2. The dynamin antibodies were from: Dynamin I—in‐house YF2 antibodies, raised in rabbits against two different synthetic peptides: dynamin I 629‐647 (RVGDKEKASETEENGSDSF) and dynamin I 765–778 (VQSVPAGRRSPTSS), used at 1/2000 dilution, as previously described 53; Dynamin II—Santa Cruz (C‐18, sc‐6400, used at 1/1000) and Dynamin III—Santa Cruz (P‐13, sc‐69472, used at 1/500 dilution).

### Animals

All animal work performed was carried out according to institutional and national care and ethics guidelines.

### Chemical synthesis

All starting materials are described in Supporting Information. ^1^H and ^13^C spectra were recorded on a Bruker Advance AMX 300 MHz spectrometer at 300.13 and 75.48 MHz or a Bruker Ascend™ 400 MHz spectrometer at 400 and 100 MHz, respectively. Microanalyses were performed at MicroAnalytical Unit, Research School of Chemistry at The Australian National University, Canberra. For the synthesis of **4a** (3‐hydroxynaphthalene‐2‐carboxylic acid (2,4,5‐trihydroxybenzylidene) hydrazide), a solution of 3‐hydroxy‐2‐naphthoic hydrazide (0.2022 g, 0.1 mmol), 2,4,5‐trihydroxybenzaldehyde (0.1540 g, 1 mmol) and ethanol (25 mL) were heated to reflux for 2 h. After this time, the reaction mixture was allowed to cool and the solvent removed *in vacuo*. The resulting solid was recrystallized from ethanol [0.162 g (45%)]. Details on yield, analysis, purity and synthesis of the other Dyngo compounds are provided in Supporting Information.

### Compounds for screening

Compounds were made as stock solutions in 100% dimethyl sulfoxide (DMSO), which were stored frozen. These were diluted in 50% v/v DMSO/20 mM Tris–HCl pH 7.4 or cell media prior to a second dilution in the aqueous assay immediately prior to use. Compounds in aqueous solutions were not exposed to temperature above 4°C prior to use in the assay.

### Dynamin GTPase assay

The Malachite Green colorimetric GTPase assay was as described 10. Dynamin I activity was measured in its SAI activity state or was stimulated by three different methods. As each stimulus activates dynamin to different extents, each assay required different dynamin concentrations. First, maximal dynamin activity was stimulated by sonicated PS liposomes 10. Purified dynamin I (10–20 nM, diluted in: 6 mM Tris–HCl, 20 mM NaCl and 0.01% Tween 80, pH 7.4) was incubated in 96‐well plates in GTPase buffer (5 mM Tris–HCl, 10 mM NaCl, 2 mM Mg^2+^, 0.05% Tween 80, pH 7.4, 1 µg/mL leupeptin and 0.1 mM PMSF) and GTP 0.3 mM in the presence of test compound for 30 min at 37°C in a final assay volume of 150 μL. Reactions were terminated with 10 μL of 0.5 M ethylenediaminetetraacetic acid (EDTA) pH 7.4 and Malachite Green solution (40 μL: 2% w/v ammonium molybdate tetrahydrate, 0.15% w/v malachite green and 4 M HCl) was added for 5 min. Second, dynamin (20 nM) was stimulated by 10 µg/mL of taxol‐stabilized preformed bovine brain microtubules (Cytoskeleton, Inc) using the same protocol. Third, dynamin I (50 nM) was stimulated by 1 μM of recombinant growth factor receptor‐bound protein 2 (grb2), a SH3 (Src homology)‐containing protein that stimulates dynamin about 5–10 times less efficiently than liposomes or microtubules 54. The assay conditions were as described above. Finally, dynamin (500 nM) SAI activity was measured using high concentrations of dynamin, which promote its cooperative self‐assembly into rings (but not helices) 26. The final DMSO concentration in the GTPase or endocytosis assays was at most 3.3 or 1%, respectively, but typically was at 1%. The GTPase assay for dynamin I was unaffected by DMSO up to 3.3%. Compounds were dissolved as 30 mM stocks in 100% DMSO. These stock solutions can be stored at −20°C for several months. Compounds were subsequently diluted into solutions of 50% DMSO made up in 20 mM Tris–HCl pH 7.4 and diluted again into the final assay. For analysis of the kinetics of **4a** inhibition, dynamin I at a final concentration of 17 nM was incubated with GTPase buffer containing PS (2 µg/mL) and varying amounts of GTP (50–250 μM) in the presence of **4a** at a concentration range between 0.5 and 6 μM. The reaction was stopped after 30 min by addition of EDTA (0.5 mM, pH 7.4). Curves were generated using the Michaelis–Menten equation *v* = *V*_max_[S]/(*K*_m_ + [S]), where S is the GTP substrate. After the *V*_max_ and *K*_m_ values were determined, the data were transformed using the Lineweaver–Burke equation, 1/*v* = 1/*V*_max_ + (*K*_m_/*V*_max_)(1/[S]).

### Dynamin II GTPase assay

Assay conditions were based on the dynamin I assay but contained modifications. Recombinant dynamin II was used at 50 nM, stimulated by 10 µg/mL PS. The GTPase reaction was allowed to occur for 90 min at 37°C before termination.

### Plasma protein binding

Plasma protein binding was estimated using an immobilized human serum albumin column (ChromTech Chiral‐HSA, 50 × 3.0 mm, 5 µm) with gradient elution based on a previously published method 56.

### Cell‐based endocytosis

Quantitative analysis of the inhibition of Alexa 594‐Tfn endocytosis in U2OS cells was performed on large numbers of serum‐starved cells as described 10. Synaptic vesicle recycling in cultured CGNs was monitored using FM1‐43 as described 57 (see Supporting Information). Dynamin‐independent endocytosis was measured using internalization of CT in NIH3T3 cells (using Tfn as a control) as described previously 58 with minor changes (see Supporting Information). The average number of cells for each data point was ˜1200. IC_50_ values were calculated using GraphPad Prism 5 and data were expressed as mean ± 95% confidence interval (CI) for three wells and ˜1200 cells.

### Endocytosis in synaptosomes

Highly purified synaptosomes, prepared from the cerebrum of adult male Sprague‐Dawley rats, were used to measure SVE and ADBE 59. Synaptosomes were allowed to attach to 96‐well glass‐bottom plates and endocytosis of FM4‐64 was measured after 2‐min KCl depolarization (described previously) 35. Fluorescent images of synaptosomes were acquired using an ImageXpress Micro system with a 20× air objective at excitation 476–524 nm and emission at 608–742 nm. Images were analyzed using MetaXpress software and fluorescence intensity values were normalized to control uptake of dye (set at 1.0).

### Primary neuronal cultures

Granule neuron cultures were prepared from the cerebella of 7‐day‐old rat pups as previously described 57. In all experiments, neurons were used between 8 and 10 days *in vitro*.

### Glutamate release from synaptosomes

Release of glutamate from synaptosomes was performed as described previously 60, with minor changes. The assay was carried out at 37°C in a Perkin Elmer LS50 fluorimeter. Control (1% DMSO) and **4a**‐treated samples were incubated in the presence of the compound for 30 min prior to stimulation of glutamate release. For each sample, 1 mg of crude P2 synaptosomes was diluted in 2 mL of HEPES‐buffered Krebs‐like buffer, either containing 1 mM EGTA (zero Ca^2+^) or 1 mM EGTA plus 3 mM Ca_2_Cl (2 mM free Ca^2+^). NAPD^+^ (3 mM) and glutamic acid dehydrogenase (50 U) were added and allowed to incubate for 15 min to allow residual glutamate in the sample to be metabolized. 4‐AP (3 mM) was subsequently added to stimulate glutamate release, which was recorded over a 5‐min period. Readings of fluorescence were taken every 2 seconds. All treatment conditions were carried out in zero Ca^2+^ and 2 mM Ca^2+^ conditions with the zero Ca^2+^ measurement subtracted from the 2 mM measurement for each time point. At each time point, the average control value was then subtracted to demonstrate activity‐dependent rundown of glutamate release. Each treatment was carried out in duplicate within a single experiment and each independent experiment was carried out three times on separate days using synaptosomes prepared from fresh rat brain tissue (*n* = 2 independent experiments).

### Fluorescence imaging of SV turnover using FM1‐43

Neuronal cultures were removed from culture medium and left for 10 min in incubation medium [170 mM NaCl, 3.5 mM KCl, 0.4 mM KH_2_PO_4_, 20 mM TES (*N*‐tris[hydroxy‐methyl]‐methyl‐2‐aminoethane‐sulfonic acid), 5 mM NaHCO_3_, 5 mM glucose, 1.2 mM Na_2_SO_4_, 1.2 mM MgCl_2_ and 1.3 mM CaCl_2_, pH 7.4]. Cultures were then mounted in a Warner imaging chamber (RC‐21BRFS). Invaginating membrane was loaded with FM1‐43 (10 μM) by evoking SV turnover with a brief train of action potentials (80 Hz for 10 seconds, 100 mA and 1‐millisecond pulse width, delivered using platinum wires embedded in the imaging chamber). Dye kept present for 1 min after stimulation to ensure all retrieving membrane was labeled (S1 loading). After a 10‐min rest period, accumulated dye was unloaded from nerve terminals using two consecutive maximal stimuli with incubation media supplemented with 50 mM KCl (50 mM NaCl removed to maintain osmolarity). The fluorescence decrease due to dye loss provides an estimate of the total number of synaptic vesicles turned over during stimulation (S1). After a 20‐min rest period the S1 protocol was repeated (S2 loading and unloading). Thus, for any selected nerve terminal, the S2 response has a matched individual internal control (S1). Dyngo compound **4a** (30 μM) was present for 15 min prior to and including either, S2 loading (to monitor effects on endocytosis) or S2 unloading (exocytosis). Results are represented as either cumulative histograms (S2/S1) for individual nerve terminals or averaged data (average ΔS2/ΔS1). Dye unloading was visualized using a Nikon Diaphot‐TMD epifluorescence microscope and 20× air objective at 480‐nm excitation and >510‐nm emission. Images were visualized using a Hamamatsu Orca‐ER CCD digital camera and offline imaging software (Simple PCI, Compix Imaging Systems). At least 70 nerve terminals were selected for each experiment and at least three independent experiments for each experimental condition.

### Fluorescence imaging of dextran uptake

Uptake of tetramethyrhodamine‐dextran (40 kDa) into nerve terminals of CGNs was monitored as described previously 40. Briefly, cells were left for 10 min in incubation medium and then stimulated with a train of 800 action potentials (80 Hz for 10 seconds) in the presence of tetramethyrhodamine‐dextran (50 μM). Dyngo compound **4a** (30 μM) was present for 15 min prior to and including action potential stimulation. Dextran loading was determined by the number of fluorescent puncta in a defined field of view (130 × 130 nm) using a 20× air objective at 550‐nm excitation and >575‐nm emission. Threshold analysis was performed to discount regions too large to represent individual nerve terminals (diameter greater than 2 nm). The number of dextran puncta per field for each experiment (usually 10 fields of view per experiment) was averaged and subtracted from background fluorescence. To ensure that the density of nerve terminals was consistent between fields and experimental conditions, experiments were performed on the same set of cultures.

### Labeling of endocytosis pathways by horseradish peroxidase

Granule neurons were processed for electron microscopy as previously described 40. Briefly, cells were transferred to incubation medium for 10 min and subsequently incubated with or without 30 μM **4a** for 15 min. Cultures were next stimulated with 800 action potentials (80 Hz) in incubation medium supplemented with HRP (10 mg/mL) in the presence or absence of **4a**. Cells were fixed in a 2% solution of glutaraldehyde in phosphate‐buffered saline for 30 min at 37°C directly after stimulation. After washing with 100 mM Tris (pH 7.4) cells were exposed to 0.1% diaminobenzidine and 0.2% H_2_O_2_ in 100 mM Tris. On development of color, cells were washed with 100 mM Tris and subsequently stained with 1% osmium tetroxide for 30 min. After washing, cells were post‐stained with 2% uranyl acetate for 15 min, then dehydrated using ethanol series and polypropylene oxide and embedded using Durcupan. Samples were sectioned, mounted on grids and viewed using a FEI Tecnai 12 transmission electron microscope. Intracellular structures that were less than 100 nm in diameter were arbitrarily designated to be synaptic vesicles, whereas larger structures were designated to be endosomes.

### Capacitance measurements of endocytosis at the Calyx of Held

Capacitance measurements at the Calyx of Held methods were previously described 62. C57/SV129 mixed background mice (7–10 days old) were decapitated. Transverse slices of 200‐µm thick were cut from the auditory brainstem with a vibratome. Recordings were made at room temperature in a solution that pharmacologically isolated Ca^2+^ currents. This solution contained (in mM) 105 NaCl, 20 TEA‐Cl, 2.5 KCl, 1 MgCl_2_, 2 CaCl_2_, 25 NaHCO_3_, 1.25 NaH_2_PO_4_, 25 dextrose, 0.4 ascorbic acid, 3 myo‐inositol, 2 sodium pyruvate and 0.001 tetrodotoxin (TTX), pH 7.4, when bubbled with 95% O_2_/5% CO_2_. The presynaptic pipette (3–5 MΩ) solution contained (in mM) 125 Cs‐gluconate, 20 CsCl, 4 Mg‐ATP, 10 Na_2_‐phosphocreatine, 0.3 GTP, 10 HEPES and 0.05 BAPTA, pH adjusted to 7.2 with CsOH. Presynaptic whole‐cell recordings were made with an EPC‐9 amplifier (HEKA Electronics). The series resistance (<20 MΩ) was compensated by 60%. Holding potential was −80 mV. Currents were low‐pass filtered at 5 kHz and digitized at 20 kHz.

The membrane capacitance was measured with the EPC‐9 amplifier together with the software lock‐in amplifier (PULSE, HEKA Electronics) before and after 20‐millisecond depolarization to assay the corresponding exocytosis and endocytosis. A sinusoidal stimulus was applied in addition to the DC holding potential (−80 mV). The peak‐to‐peak voltage of the sine wave was 10 mV to avoid activation of the Ca^2+^ currents. The resulting current was processed via the Lindau–Neher technique 44 to give estimates of the membrane capacitance, membrane conductance and the series conductance. The sine wave frequency was 1000 Hz. The reversal potential of the measured DC current was assumed to be 0 mV 44. During step depolarization the capacitance was not measured. The capacitance jump was measured as the difference between the averaged capacitance value in 0.4 seconds after stimulation and the baseline value. The capacitance jump returned to the baseline in a few seconds to tens of seconds. The interval between two voltage commands was at least 2 min to complete readily releasable pool replenishment and avoid short‐term synaptic plasticity induced by the previous voltage command. All capacitance traces shown in the figures were taken from single recordings and were low‐pass filtered at 200 Hz. Dyngo compound **4a** or dynasore was applied extracellularly via local puffing. We positioned glass pipettes (1.5–2.5 MΩ) containing 0.3 mM **4a** or 0.8 mM dynasore plus the bath solution described below close (<5 µm) to the surface of postsynaptic cells. The **4a** or dynasore solutions were pressure injected (4 psi) onto the surface of the synapse with a pneumatic picopump (Picospitzer III, Parker Hannifin Co).

### Dynamin‐independent CT endocytosis

Internalization of CT and Tfn in NIH3T3 cells was performed as described previously 58 with minor changes. Uptake of 5 µg/mL Tfn‐488 (Invitrogen) and 2 µg/mL CT‐555 (Invitrogen) was carried out constitutively at 37°C in serum‐free DMEM (Gibco) for 5 min. Cells were washed 2 × 1 min with glycine pH 2.2 to remove any cell surface labeling of CT 64. Cells were fixed in 4% PFA and imaged with a 510 Meta Zeiss confocal microscope. Calculation of CT‐555 and Tfn‐488 fluorescence intensity was as described 58. In brief, images were processed in Adobe Photoshop CS2. All images were captured under the same detection settings and a threshold was applied. The mean pixel intensity and number of pixels obtained from the histogram were used to calculate total fluorescence intensity per cell. Control cells were normalized to 100% and each sample was recalculated in relation to control cells to give fluorescence intensity as a percentage of control cells.

All other methods are described in Supporting Information.

## Supplementary Material

Table S1. MTT analysis of different human cancer cell lines after 72 h of incubation with Dyngo analogs. GI_50_ (μM) is the concentration that inhibits cell growth by 50% (the lower the value the greater the growth inhibition). Errors represent SEM (n = 3 independent experiments).

Appendix S1. Dyngo library 2.

**Scheme S1. Scheme for the synthesis of Dyngo analogs.**

**Figure S1. Dynasore is a poor dynamin I inhibitor when assayed in the presence of Tween‐80.** A) Structure of dynasore. B) Dose‐dependent inhibition by dynasore of dynamin I GTPase activity stimulated by PS liposomes in the presence of Tween‐80. C) IC_50_ values of dynamin I after activation by four mechanisms in the presence of Tween‐80. Dynasore was either produced in house (synthesized), purchased from Sigma or obtained from the laboratory of Tom Kirchhausen (TK). Dynasore was tested at a range of concentrations up to a maximum of 1 mM, with the exception of data marked with *, which were tested up to 1.5 mM. D) Effect of dynasore on endocytosis of Tfn‐A594 in U2OS cells. All data are means ± SEM of three independent experiments.

**Figure S2. Dyngo compound 4a has no effect on dynamin binding to SH3 domains.** Pull down of dynamin I in the absence or presence of the indicated **4a** concentrations was performed using the SH3 domains of Grb2, endophilin I or amphiphysin I attached to GSH beads. The proteins were resolved on 12% SDS‐PAGE gels and visualized using Coomasie Blue. The results are shown for one experiment performed in triplicate and the same results were obtained in two further independent experiments (in duplicate).

**Figure S3. Dyngo compounds do not affect amphiphysin protein–protein interactions.** The effect of dynasore and Dyngo compounds on binding of clathrin heavy‐chain C‐terminal domain or AP‐2 alpha ear domain to amphiphysin 1 PRD + CLAP domains determined by ELISA assays. Data are mean and error bars represent SEM for triplicate measurements for n = 1.

**Figure S4. Dyngo series 4a, 6a and dynasore are non‐toxic and do not affect cell viability in HeLa cells.** A and B) HeLa cells were exposed to MiTMAB or the indicated Dyngo compound for 8 h in the presence (A) and absence of serum (B) and then analyzed using an LDH assay. Data represent SEM (n = 2 independent experiments). C–F) Cell membrane integrity as an indicator of viability (C and E) and cell proliferation (D and F) in HeLa cells were analyzed after prolonged exposure (20 h) to **4a**, **6a** and dynasore in the presence (C and D) and absence of serum (E and F) using a trypan blue exclusion assay. Data represent SEM (n = 2 independent experiments).

**Figure S5. Effect of dynasore analogs on mitochondria in HeLa cells.** A) HeLa cells stably expressing H2B‐mCherry (red) were serum‐starved, incubated with Mitotracker Green FM (green) and imaged by confocal microscopy. The left panel shows cells at 40× magnification, while the right panel shows greater detail of mitochondrial structure. All nuclei exhibited red fluorescence, although the intensity varied considerably. Cells were then treated with either DMSO (B), 30 μM **4a** (C), 100 μM dynasore (D) or 30 μM **6a**. In (B) to (E), left‐hand panels show images acquired 30 min after treatment, central panels show a more detailed image of mitochondria after 30 min of treatment and the right‐hand panels show the cells after 60 min. After 30 min of treatment, **4a**‐ and dynasore‐treated cells exhibited unchanged mitochondrial morphology, including elongated mitochondria (arrows in A–D), while **6a**‐treated cells exhibited relatively fragmented mitochondria (arrows in E). After 60 min of treatment, all treated cells exhibited a reduction in Mitotracker Green FM fluorescence. Scale bars = 20 µm for images in left‐ and right‐hand panels, while for zoomed panels the scale bar = 5 µm.

**Figure S6. U2OS cells express only dynamin II.** Equal protein load (50 µg) from four different cancer cell lines was run on SDS gels along with 0.2 µg partially purified full‐length recombinant dynamin I, II or III. The three dynamins were detected with isoform‐specific antibodies by western blot. Results shown are for one experiment with duplicate or triplicate cell samples and similar results were obtained in two additional experiments.

**Figure S7. Dyngo compound 4a does not block dynamin‐independent endocytosis of cholera toxin.** A) NIH3T3 cells were serum starved for 3 h in unsupplemented DMEM. Cells were subsequently pretreated (or not) for 20 min with 20, 50 or 80 μM **4a** or dynasore. Cells were next incubated with 5 µg/mL Tfn‐488 and 2 µg/mL CT‐555 in the continued presence of 20, 50 or 80 μM **4a** or dynasore for 5 min at 37°C. 2 × 1‐min washes with 0.5 M glycine and pH 2.2 were performed to remove surface labeling of CT‐555 prior to fixation in 4% paraformaldehyde. Cells were imaged on a 510 Meta Zeiss confocal microscope. Scale bar is 10 µm. B) Over 50 cells treated with each condition in (A) were imaged and fluorescence intensity was calculated based on each unique histogram profile. Each treated sample was calculated as a percentage of control units. All data are means ± SEM.

**Figure S8. Blockade of synaptic vesicle turnover in CGNs.** A–D) Activity‐dependent loading and unloading of the styryl dye FM1‐43 in CGN cultures. Representative images either loaded at S1 (A) or S2 (B) in the absence of **4a** or in its presence (S1—panel C; S2—panel D) are displayed. Scale bar represents 1 µm. E and F) Dextran endocytosis specifically reflects ADBE relative to CME. Representative images of control (E) or **4a** (panel F, 30 μM, 15‐min preincubation) inhibition of uptake of fluorescent tetramethyrhodamine‐dextran (50 μM) in CGNs electrically stimulated by a train of 800 action potentials (80 Hz) followed by immediate dextran washout.

**Figure S9. Dynasore inhibits SVE in neurons.** The effect of dynasore on whole‐cell membrane capacitance was investigated at the Calyx of Held in parallel with the experiments in Figure 4 except that there was 0.8 mM dynasore in the puffing pipette. A) A sample trace shows membrane capacitance on control (black) and dynasore‐treated samples (red). B) Collated data of normalized capacitance measurement from control and dynasore‐treated neurons (n = 7).
