# Supplementary material for: Building a Better Dynasore: The Dyngo Compounds Potently Inhibit Dynamin and Endocytosis
Source: Traffic. 2013 Oct 9;14(12):1272–89. doi: 10.1111/tra.12119 (PMC4138991; doi:10.1111/tra.12119)
Supplement: Supplementary file 1 — Table S1. MTT analysis of different human cancer cell lines after 72 h of incubation with Dyngo analogs. GI50 (μM) is the concentration that inhibits cell growth by 50% (the lower the value the greater the growth inhibition). Errors represent SEM (n = 3 independent experiments). [file tra-14-1272-s1.docx]

**SUPPLEMENTARY TABLE**

**Table S1**: MTT analysis of different human cancer cell lines after 72 h of incubation with *Dyngo* analogues. GI_50_ (μM) is the concentration that inhibits cell growth by 50% (the lower the value the greater the growth inhibition). Errors represent SEM (n=3 independent experiments).

|  | GI_50_ (µM) | | |
| --- | --- | --- | --- |
|  | **4a** | **6a** | Dynasore |
| HT29 (Colon) | > 50 | > 50 | 12 ± 1.3 |
| SW480 (Colon) | > 50 | > 50 | 3.6 ± 0.1 |
| MCF-7 (Breast) | > 50 | > 50 | 14 ± 0.3 |
| A42780 (Ovarian) | > 50 | > 50 | 6.7 ± 0.4 |
| H460 (Lung) | > 50 | > 50 | 21 ± 1.9 |
| A431 (Skin) | > 50 | > 50 | 14 ± 0.6 |
| DU145 (Prostate) | > 50 | > 50 | 23 ± 1.7 |
| BE2-C (Neuroblastoma) | > 50 | > 50 | 8.6 ± 0.4 |
| SJ-G2 (Glioblastoma) | > 50 | > 50 | 12 ± 1.7 |
