# Supplementary material for: Building a Better Dynasore: The Dyngo Compounds Potently Inhibit Dynamin and Endocytosis
Source: Traffic. 2013 Oct 9;14(12):1272–89. doi: 10.1111/tra.12119 (PMC4138991; doi:10.1111/tra.12119)
Supplement: Supplementary file 2 — Appendix S1. Dyngo library 2. [file tra-14-1272-s2.docx]

Building a Better Dynasore:

The Dyngo Compounds Potently Inhibit Dynamin and Endocytosis

Adam McCluskey^1*^, James A. Daniel^2*^, Gordana Hadzic^1^, Ngoc Chau^2^, Emma L. Clayton^3^, Anna Mariana^2^, Ainslie Whiting^2^, Nick Gorgani^2^, Jonathan Lloyd^2^, Annie Quan^2^, Lia Moshkanbaryans^2^, Sai Krishnan^2^, Swetha Perera^2^, Megan Chircop^2^, Lisa von Kleist^10^, Andrew B. McGeachie^4^, Mark T. Howes^5^, Robert G. Parton^5^, Michael Campbell^6^, Jennette A. Sakoff^7^, Xuefeng Wang^8^, Jian-Yuan Sun^8^, Mark J. Robertson^1^, Fiona M. Deane^1^, Tam H. Nguyen^9^, Frederic A. Meunier^9^, Michael A. Cousin^3^, and Phillip J. Robinson^2^

^1^ Chemistry, Centre for Chemical Biology, School of Environmental and Life Sciences, The University of Newcastle, Callaghan, NSW 2308, Australia.

^2^ Cell Signalling Unit, Children’s Medical Research Institute, The University of Sydney

Locked Bag 23, Wentworthville, Sydney, NSW 2145, AUSTRALIA,

Tel: +61-2-9687-2800; Fax: +61-2-9687-2120.

^3^ Centre for Integrative Physiology, Hugh Robson Building, University of Edinburgh, Edinburgh, EH8 9XD.

^4^ Neuroscience Research Australia, Hospital Road, Randwick NSW 2031, Australia

^5^ Institute for Molecular Bioscience and Centre for Microscopy and Microanalysis, University of Queensland, Brisbane, Queensland 4072, Australia.

^6^ Centre for Drug Candidate Optimisation, Monash Institute of Pharmaceutical Sciences, Monash University, Parkville, Victoria 3052, Australia.

^7^ Department of Medical Oncology, Calvary Mater Newcastle Hospital, Edith Street, Waratah, NSW 2298, Australia.

^8^ Jian-Yuan Sun Institute of Biophysics, Chinese Academy of Sciences.

^9^ Queensland Brain Institute, The University of Queensland, Brisbane, Queensland, 4072, Australia.

^10^ Department of Membrane Biochemistry, Institute of Chemistry and Biochemistry, Freie Universität Berlin, 14195, Berlin, Germany

^*^ These authors contributed equally to this work

**Synopsis**

Dynamin is essential for clathrin-mediated endocytosis (CME). We describe the Dyngo^TM^ series of dynamin inhibitors with greatly improved potency, reduced cytotoxicity and reduced detergent binding compared to their parent, dynasore. Dyngo compound **4a** is 37-fold more potent for dynamin inhibition and at least six-fold more potent for CME inhibition in cells and nerve terminals. This series are the first to target one conformational state of dynamin, inhibiting lipid-associated helical dynamin but not dynamin rings induced by self-assembly or SH2-domain-containing proteins.

*Corresponding author:*

Phillip J. Robinson

Cell Signalling Unit,

Children’s Medical Research Institute,

Locked Bag 23, Wentworthville,

Sydney, NSW 2145, AUSTRALIA,

Tel: +61-2-9687-2800

Fax: +61-2-9687-2120

E-mail: probinson@cmri.org.au

**SUPPLEMENTARY INFORMATION – DYNGO LIBRARY 2**

Dyngo Library 2 (Table 1) were designed using a combination of molecular modelling studies to ensure complementarity of electrostatics and bioisosteric replacements to evaluate the role of the di- or tri-hydroxy phenyl moieties in the non-specific binding of the compounds. We firstly developed Dyngo analogues **10a** and **11a** to confirm the –OH moiety requirement for dynamin activity. As predicted, they displayed markedly reduced dynamin inhibition with **11a** being the most active (IC_50_ = 58.9 and 44.2 μM, Tween and Tween-free respectively), supporting the notion that an –OH moiety confers both activity and Tween sensitivity. Next we developed the C3′,C4′ bioisosteric dynasore analogues **12a**, **13a** and **14a**. These analogues were about equipotent with dynasore and showed no major potency differences when assayed in the presence or absence of Tween-80. The reason for the lack of CME inhibition associated with **12a**, **13a** and **14a** is unknown, but might be a consequence of factors such as modified membrane permeability.

**SUPPLEMENTARY MATERIALS AND METHODS**

**Biology**

**Pull-down experiments**

Recombinant human dynamin I was expressed in insect cells (SF21). Cells were lysed and sonicated in 20 mM HEPES buffer with 250 mM NaCl. The lysates were diluted in 20 mM HEPES buffer alone to obtain a final 50 mM NaCl concentration. This diluted lysate was used for pull-downs with 6 µg of GST-amphiphysin1 SH3, GST-endophilin SH3 or GST-grb2 SH3 (all recombinantly expressed in bacteria) immobilised on glutathione sepharose beads. Incubations were done in the presence or absence of 10, 40 and 100 µM **4a** for 1 hr at 10°C. The GSH beads were then washed three times with ice cold PBS and the bound proteins were separated on 12% SDS-PAGE gels and visualised using Coomassie Blue.

**Lactate dehydrogenase (LDH) toxicity assay**

Cytotoxicity was assayed by determination of lactate dehydrogenase (LDH) activity. HeLa cells were seeded in 96 well plates as described previously (11) Asynchronously growing cells were treated in the presence or absence of MiTMAB or the indicated Dyngo compound in the presence and absence of FCS at the indicated concentration for 8h. The supernatant (50 μL) was added to 100 μL of LDH assay reagent (Sigma- Aldrich) and the reaction was allowed to develop for 20 min. Absorbance was measured at 490 nm and 690 nm (plate background absorbance). Values were normalized to drug/media background value and toxicity was calculated as a % of a 100% lysed cell control.

**Cell viability and proliferation assay**

Cell viability and membrane integrity was assessed by the Trypan Blue exclusion assay. Cells were seeded into 10 cm dish at a density of 1 × 10^5^ cells per dish. On day 0 (24 h after seeding), cells were treated in the presence and absence of the indicated Dyngo compound or control (0.5% DMSO) for 20 h. This was carried out in the presence and absence of FBS. Cells (floating and adherent) were collected immediately prior to drug treatment as well as after the 20 h treatment and cell number and viability were measured using a Vi-CELL XR cell viability analyzer 2.03 (Beckman Coulter) as previously described (11,34).

***In vitro* growth inhibition assay (MTT assay)**

A 30 mM stock solution of each test compound was prepared in dimethylsulphoxide (DMSO) and stored at −20 °C. All cell lines were cultured at 37 °C, under 5% CO_2_ in air and were maintained in Dulbecco’s modified Eagle’s medium (Trace Biosciences, Australia) supplemented with 10% foetal bovine serum, 10 mM sodium bicarbonate penicillin (100 IU/mL), streptomycin (100 µg/mL), and glutamine (4 mM). Cells in logarithmic growth were transferred to 96-well plates. Cytotoxicity was determined by plating cells in duplicate in 100 mL medium at a density of 2,500–4,000 cells/well. On day 0, (24 h after plating) when the cells were in logarithmic growth, 100 μL medium with or without the test agent was added to each well. After 72 h drug exposure, growth inhibitory effects were evaluated using the MTT (3-[4,5-dimethyltiazol-2-yl]-2,5-diphenyl-tetrazolium bromide) assay and absorbance read at 540 nm. A dose response curve was produced allowing for the calculation of a GI_50_ value. This value is the drug concentration at which cell growth is inhibited by 50% based on the difference between the optical density values on day 0 and those at the end of drug exposure.

**Mitotracker labeling of mitochondria**

HeLa cells were used that had been stably transduced to express H2B-mCherry. Cells were cultured at 37 °C, 5% CO_2_ in RPMI medium with 10% FBS. Expression of the transgene was maintained by puromycin selection. The day before imaging, cells were seeded on 4-chambered, glass bottomed 35 mm round dish at a density of 1 x 10^5^ cells per chamber. The next day, cells were washed 4x in PBS and then incubated either in RPMI + 10% FBS or RPMI alone for 4 h. Medium was then removed and replaced with Mitotracker green-FM (150 nM) diluted in Hank’s buffered salt solution. Cells were incubated in Mitotracker for 20 min and then returned to RPMI either with or without serum. Cells were then imaged in a Yokugawa CV1000 Cell Voyager live cell confocal system prior to the application of compounds. Compounds were then applied: control samples received 1% DMSO, while test samples received **4a**, **6a** or dynasore (final DMSO concentration of 1%). Cells were incubated for at 37 °C, 5% CO_2_ and then images acquired over 2 h at 6 min intervals. 6 images were taken of each test condition.

**ELISA-based Clathrin and AP-2 binding assays**

The assay for the interaction of amphiphysin with either clathrin or AP2 was based on our previous report (11). Bacterially expressed recombinant purified His6-tagged amphiphysin 1 PRD+CLAP domains (amino acids 250-578) in screening buffer (20 mM HEPES, pH 7.4, 50 mM NaCl, 1 mM DTT, 1 mM PMSF) was added to a 384 well ELISA plate (high-binding PS Microplate, Greiner Bio-One) and bound to the plastic at room temperature for 1 hr. Non-specific binding was reduced by overnight incubation with 50 µL blocking buffer (20 mM HEPES, pH 7.4, 50 mM NaCl, 1 mM PMSF, 2 % BSA, 2.5 % skim milk) at 4 °C. Following extensive washes (with 20 mM HEPES, pH 7.4, 50 mM NaCl, 0.05 % Tween 20), chemical compounds diluted in DMSO (10 µL) were added and incubated together with bacterially expressed recombinant GST-tagged clathrin heavy chain TD (amino acids 1-364) or GST-AP-2 α-appendage (amino acids 702–938) for 1 hr at room temperature in screening buffer. This incubation was in the presence of test compounds diluted in 1% DMSO or 1% DMSO alone as a control. After 3 washes, horseradish peroxidase-coupled anti-GST antibodies were added in screening buffer and the plate incubated for 15 min at room temperature. Following additional washes, 50 µL TMB (3,3′,5,5′-tetramethylbenzidine) (Pierce Biotechnology) chromogenic substrate of horseradish peroxidase was added and the plate was incubated for 20 min before the reaction was terminated by adding 50 µL 1 N sulfuric acid to produce a yellow colour. The amount of bound protein was determined by spectrophotometric measurement in a plate reader in triplicates. Relative binding was calculated as a percentage of the DMSO control.

**Chemistry**

*General*: All starting materials were reagent grade purchased from Sigma-Aldrich and Maybridge and utilized without further purification. All organic solvents were distilled prior to use. Organic solvent extracts were dried with MgSO_4_ and removed under reduced pressure with either Büchi or Heidolph rotary evaporators. ^1^H and ^13^C spectra were recorded on a Bruker Avance^TM^ AMX 300 MHz spectrometer at 300.13 and 75.48 MHz or a a Bruker Ascend™ 400MHz spectrometer at 400 and 100 MHz respectively. Spectra were recorded using deuterated chloroform (CDCl_3_) and deuterated dimethyl sulfoxide (DMSO-*d*_6_). For CDCl_3_ the chemical shifts are relative to 0.5% TMS (tetramethylsilane) as internal standard. For DMSO-*d*_6_, the residual solvent peaks were used as internal reference [*δ* 2.49 (quintet) and 39.7 ppm (septet) for ^1^H and ^13^C respectively]. Chemical shifts (*δ*) are reported in parts per million (ppm) measured relative to the internal standards and coupling constant (*J*) are expressed in Hertz (Hz). Multiplicities are denoted as singlet (s), broad singlet (bs), doublet (d), doublet of doublets (dd), triplet (t), doublet of triplets (dt), quartet (q), quintet (quint) and multiplet (m). Peaks are listed according to the following convention: chemical shift, integration, multiplicity, coupling constant, and assignment. Melting points were determined on a Stuart melting point apparatus (SMP11) and are uncorrected. Analytical thin layer chromatography (TLC) was carried out on Merck silica gel 60 F_254_ pre-coated aluminium plates. Visualization was achieved using UV light followed by dipping plates in a solution of phosphomolybdic acid [2.5% H_2_SO_4_, 2% H_3_PO_3_MoO_4_, 1% Ce(SO_4_)_2_] and heating. Microanalyses were performed at MicroAnalytical Unit, Research School of Chemistry, at The Australian National University, Canberra. All compounds returned satisfactory analyses.

**General procedure for the synthesis of *Dyngo* derivatives**

To a stirring solution of 3-hydroxy-2-naphthoic hydrazide (1 mmol) in ethanol (25 mL) was added the desired aldehyde (1 mmol) and the reaction mixture was heated under reflux for 2 h. The reaction mixture was allowed to cool and the resulting precipitate was isolated by suction filtration, washed with cold ether and recrystallized from ethanol to afford the desired *Dyngo* derivative. NMR spectra and HPLC chromatograms of the synthesized *dyngo* analogues were consistent with the presence of only one geometric isomer at the imine bond - the *E* configuration. However, *N*-acyl hydrazones exist in equilibrium between the 2 stable conformers, *syn-periplanar* (sp) and *anti-periplanar* (ap) in solution. These conformers are able to interconvert through rotation of the amide bond (65). Data corresponding to the major isomer, the anti-periplanar isomer, is quoted below. The minor conformer was never found to exceed 8% relative to the major conformer.

***(*E*)-*N*'-(2,4-Dihydroxybenzylidene)-3-hydroxy-2-naphthohydrazide (1a)***

Starting from 3-hydroxy-2-naphthoic hydrazide (0.202 g, 1 mmol) and 2,4-dihydroxybenzaldehyde (0.138 g, 1 mmol), **1a** (0.20 g, 62%) was obtained as an off-white solid (m.p. 293–294 °C);

^1^H NMR (400 MHz, DMSO-*d_6_*) δ 11.97 (s, 1H), 11.40 (s, 1H), 10.03 (d, *J* = 7.8 Hz, 1H), 8.57 (s, 1H), 8.47 (s, 1H), 7.91 (d, *J* = 8.1 Hz, 1H), 7.77 (d, *J* = 8.3 Hz, 1H), 7.56 – 7.49 (m, 1H), 7.42 – 7.32 (m, 3H), 6.40 (dd, *J* = 8.4, 2.3 Hz, 1H), 6.36 (d, *J* = 2.2 Hz, 1H);

^13^C NMR (101 MHz, DMSO-*d_6_*) δ 163.8, 161.4, 160.0, 154.7, 150.2, 136.3, 131.8, 130.6, 129.1, 128.7, 127.2, 126.3, 124.3, 120.2, 111.1, 111.0, 108.3, 103.1;

MS: ESI +ve = 323 (M+1);

Micro analysis - calculated for C_18_H_14_N_2_O_4_: C 67.07, H 4.38, N 8.69; found: C 67.29; H 4.57; N 8.24.

***(*E*)-*N*'-(2,4-Dihydroxy-5-methoxybenzylidene)-3-hydroxy-2-naphthohydrazide (2a)***

Starting from 3-hydroxy-2-naphthoic hydrazide (0.202 g, 1 mmol) and 3,4-dihydroxy-5-methoxybenzaldehyde (0.168 g, 1 mmol), **2a** (0.130 g, 37%) was obtained as a yellow solid (m.p. 247–248 °C);

^1^H NMR (400 MHz, DMSO-*d_6_*)) δ 11.87 (bs, 1H), 11.42 (bs, 1H), 9.27 (bs, 1H), 8.85 (bs, 1H), 8.48 (s, 1H), 8.30 (s, 1H), 7.92 (d, *J* = 8.2 Hz, 1H), 7.77 (d, *J* = 8.3 Hz, 1H), 7.52 (t, *J* = 7.5 Hz, 1H), 7.36 (m, 2H), 6.92 (d, *J* = 1.6 Hz, 1H), 6.90 (d, *J* = 1.6 Hz, 1H), 3.84 (s, 3H);

^13^C NMR (101 MHz, DMSO-*d_6_*) δ 164.1, 154.8, 149.9, 149.0, 146.4, 137.5, 136.3, 130.5, 129.1, 128.7, 127.2, 126.3, 124.9, 124.3, 120.6, 111.1, 109.1, 103.1, 56.37;

MS: ESI +ve = 353 (M+1);

Micro analysis – calculated for C_19_H_16_N_2_O_5_: C 64.77, H 4.58, N 7.95; found: C 65.12; H 4.76; N 8.21.

***(*E*)-3-Hydroxy-*N*'-(2,3,4-trihydroxybenzylidene) -2-naphthohydrazide (3a)***

Starting from 3-hydroxy-2-naphthoic hydrazide (0.202 g, 1 mmol) and 2,3,4-trihydroxybenzaldehyde (0.154 g, 1 mmol), **3a** (0.210 g, 62%) was obtained as a light brown solid (m.p. 284–285 °C);

^1^H NMR (400 MHz, DMSO-*d_6_*) δ 12.03 (bs, 1H), 11.45 (s, 2H), 9.54 (bs, 1H), 8.53 (s, 1H), 8.48 (s, 1H), 7.92 (d, *J* = 8.1 Hz, 1H), 7.77 (d, *J* = 8.3 Hz, 1H), 7.59 – 7.48 (m, 1H), 7.41 – 7.32 (m, 2H), 6.84 (d, *J* = 8.5 Hz, 1H), 6.43 (d, *J* = 8.4 Hz, 1H);

^13^C NMR (101 MHz, DMSO-*d_6_*) δ 163.8, 154.7, 151.3, 149.5, 148.1, 136.4, 133.2, 130.7, 129.13, 128.7, 127.2, 126.3, 124.3, 121.7, 120.2, 111.3, 111.1, 108.2;

MS: ESI −ve = 337 (M−1);

Micro analysis – calculated for C_18_H_14_N_2_O_5_: C 63.90, H 4.17, N 8.28; found: C 64.15; H 4.28; N 8.07.

***(*E*)-3-Hydroxy-*N*'-(2,4,5-trihydroxybenzylidene) -2-naphthohydrazide (4a)***

Starting from 3-hydroxy-2-naphthoic hydrazide (0.202 g, 1 mmol) and 2,4,5-trihydroxybenzaldehyde (0.154 g, 1 mmol), **4a** (0.160 g, 47%) was obtained as a yellow solid (m.p. 181–182 °C);

^1^H NMR (400 MHz, DMSO-*d_6_*) δ 11.97 (bs, 1H), 11.45 (bs, 1H), 10.54 (s, 1H), 9.61 (bs, 1H), 8.63 (bs, 1H), 8.52 (s, 1H), 8.49 (s, 1H), 7.91 (d, *J* = 8.3 Hz, 1H), 7.77 (d, *J* = 8.3 Hz, 1H), 7.52 (t, *J* = 7.5 Hz, 1H), 7.37 (t, *J* = 7.5 Hz, 1H), 7.33 (s, 1H), 6.96 (s, 1H), 6.38 (s, 1H);

^13^C NMR (101 MHz, DMSO-*d_6_*) δ 163.9, 154.9, 152.5, 150.1, 149.6, 139.1, 136.4, 130.4, 129.1, 128.7, 127.2, 126.3, 124.3, 120.0, 115.0, 111.1, 109.9, 103.9;

MS: ESI +ve = 339 (M+1);

Micro analysis – calculated for C_18_H_14_N_2_O_5_.0.5H_2_O: C 62.25, H 4.35, N 8.07; found: C 62.26; H 4.80; N 7.57.

***(*E*)-3-Hydroxy-*N*'-(3,4,5-trihydroxybenzylidene) -2-naphthohydrazide (5a)***

Starting from 3-hydroxy-2-naphthoic hydrazide (0.202 g, 1 mmol) and 3,4,5-trihydroxybenzaldehyde (0.154 g, 1 mmol), **5a** (0.220 g, 65%) was obtained as a yellow solid (m.p. 252–253 °C);

^1^H NMR (400 MHz, DMSO-*d_6_*) δ 11.80 (bs, 1H), 11.45 (bs 1H), 9.19 (bs, 2H), 8.48 (s, 1H), 8.21 (s, 1H), 7.91 (d, *J* = 8.2 Hz, 1H), 7.76 (d, *J* = 8.3 Hz, 1H), 7.51 (t, *J* = 7.4 Hz, 1H), 7.36 (t, *J* = 7.4 Hz, 1H);

^13^C NMR (101 MHz, DMSO-*d_6_*) δ 164.1, 162.8, 154.9, 150.0, 146.7, 136.5, 136.3, 130.4, 129.1, 128.7, 127.2, 126.3, 124.9, 124.3, 120.3, 111.1, 107.0;

MS: ESI +ve = 339 (M+1);

Micro analysis – calculated for C_18_H_14_N_2_O_5_: C 63.90, H 4.17, N 8.28; found: C 63.45; H 4.32; N 8.45.

***(*E*)-*N*'-(2,3-Dihydroxybenzylidene)-3-hydroxy-2-naphthohydrazide (6a)***

Starting from 3-hydroxy-2-naphthoic hydrazide (0.202 g, 1 mmol) and 2,3-dihydroxybenzaldehyde (0.138 g, 1 mmol), **6a** (0.191 g, 59%) was obtained as a yellow solid (m.p. 178–179 °C);

^1^H NMR (400 MHz, DMSO-*d_6_*) δ 12.16 (bs, 1H), 11.30 (bs, 1H), 11.08 (s, 1H), 9.28 (s, 1H), 8.66 (s, 1H), 8.48 (s, 1H), 7.93 (d, *J* = 8.2 Hz, 1H), 7.78 (d, *J* = 8.3 Hz, 1H), 7.57 – 7.48 (m, 1H), 7.42 – 7.32 (m, 2H), 7.02 (dd, *J* = 7.8, 1.3 Hz, 1H), 6.90 (dd, *J* = 7.8, 1.4 Hz, 1H), 6.77 (dd, *J* = 7.8, 7.8 Hz, 1H);

^13^C NMR (101 MHz, DMSO-*d_6_*) δ 164.0, 154.5, 150.0, 146.7, 146.1, 145.5, 136.4, 130.9, 129.2, 128.8, 127.3, 126.3, 124.3, 120.5, 119.7, 119.3, 118.0, 111.1;

MS: ESI +ve = 323 (M+1);

Micro analysis – calculated for C_18_H_14_N_2_O_4_.0.75H_2_O: C 64.38, H 4.65, N 8.34; found: C 64.09; H 4.27; N 8.24.

***(*E*)-*N*'-(3,4-Dihydroxybenzylidene)-3-hydroxy-2-naphthohydrazide (dynasore)***

Starting from 3-hydroxy-2-naphthoic hydrazide (0.202 g, 1 mmol) and 3,4-dihydroxybenzaldehyde (0.138 g, 1 mmol), dynasore (0.172 g, 53%) was obtained as a yellow solid (m.p. 155–156 °C);

^1^H NMR (400 MHz, DMSO-*d_6_*) δ 11.85 (bs, 1H), 9.38 (bs, 2H), 8.48 (s, 1H), 8.30 (s, 1H), 7.92 (d, *J* = 8.1 Hz, 1H), 7.77 (d, *J* = 8.3 Hz, 1H), 7.57 – 7.47 (m, 1H), 7.41 – 7.33 (m, 2H), 7.32 (d, *J* = 1.9 Hz, 1H), 7.02 (dd, *J* = 8.2, 1.9 Hz, 1H), 6.84 (d, *J* = 8.1 Hz, 1H);

^13^C NMR (101 MHz, DMSO-*d_6_*) δ 164.2, 154.9, 149.7, 148.7, 146.2, 136.3, 130.4, 129.1, 128.7, 127.2, 126.3, 126.0, 124.2, 121.4, 120.3, 116.1, 113.4, 111.1;

MS: ESI +ve = 323 (M+1);

Micro analysis – calculated for C_18_H_14_N_2_O_4_.H_2_O: C 63.52, H 4.74, N 8.23; found: C 64.09; H 4.27; N 8.24.

***(*E*)-*N*'-(3,5-Dihydroxybenzylidene)-3-hydroxy-2-naphthohydrazide (8a)***

Starting from 3-hydroxy-2-naphthoic hydrazide (0.202 g, 1 mmol) and 3,5-dihydroxybenzaldehyde (0.138 g, 1 mmol), **8a** (0.162 g, 50%) was obtained as a yellow solid (m.p. 305–306 °C);

^1^H NMR (400 MHz, DMSO-*d_6_*) δ 11.93 (bs, 1H), 9.54 (bs, 2H), 8.47 (s, 1H), 8.29 (s, 1H), 7.91 (s, *J* = 8.1 Hz, 2H), 7.76 (d, *J* = 8.3 Hz, 1H), 7.51 (t, *J* = 7.5 Hz, 1H), 7.40 – 7.30 (m, 2H), 6.68 (s, 2H), 6.32 (s, 1H);

^13^C NMR (101 MHz, DMSO-*d_6_*) δ 164.3, 162.8, 154.7, 149.4, 136.3, 136.3, 130.7, 129.1, 128.7, 127.2, 126.3, 124.3, 120.7, 111.1, 105.9, 105.2**;**

MS: ESI +ve = 323 (M+1);

Micro analysis – calculated for C_18_H_14_N_2_O_4_: C 67.07, H 4.38, N 8.69; found: C 67.47; H 4.52; N 8.75.

***(E)-3-hydroxy-N'-((1-methyl-1H-indol-3-yl)methylene)-2-naphthohydrazide (9a)***

Starting from 3-hydroxy-2-naphthoic hydrazide (0.202 g, 1 mmol) and 1-methylindole-2-carboxaldehyde (0.159 g, 1 mmol), **9a** (0.150 g, 44%) was obtained as a pale yellow solid (m.p. 261–262 °C);

^1^H NMR (400 MHz, DMSO-*d_6_*) δ 11.82 (bs, 2H), 8.64 (s, 1H), 8.52 (s, 1H), 8.34 (d, *J* = 7.7 Hz, 1H), 7.91 (d, *J* = 8.2 Hz, 1H), 7.87 (s, 1H), 7.77 (d, *J* = 8.3 Hz, 1H), 7.52 (m, 2H), 7.41 – 7.19 (m, 5H), 3.85 (s, 3H);

^13^C NMR (101 MHz, DMSO-*d_6_*) δ 164.1, 155.4, 145.9, 138.1, 136.3, 134.9, 130.0, 129.1, 128.6, 127.2, 126.3, 125.2, 124.2, 123.3, 122.6, 121.3, 120.0, 111.1, 111.0, 110.7;

MS: ESI +ve = 344 (M+1);

Micro analysis – calculated for C_21_H_17_N_3_O_2_: C 73.45, H 4.99, N 12.24; found: C 73.90; H 5.11; N 12.69.

***(E)-N-(4-{[2-(3-hydroxy-2-naphthoyl)hydrazono]methyl}phenyl)acetamide (10a)***

Starting from 3-hydroxy-2-naphthoic hydrazide (0.202 g, 1 mmol) and 4-acetamidobenzaldehyde (0.163 g, 1 mmol), **10a** (0.161 g, 46%) was obtained as a pale yellow solid (m.p. 291–292 °C);

^1^H NMR (400 MHz, DMSO-*d_6_*) δ 11.93 (bs, 1H), 11.40 (bs, 1H), 10.17 (s, 1H), 8.47 (s, 1H), 8.41 (s, 1H), 7.92 (d, *J* = 8.1 Hz, 1H), 7.77 (d, *J* = 8.3 Hz, 1H), 7.71 (s, 4H), 7.58 – 7.48 (m, 1H), 7.36 (m, 1H), 7.33 (s, 1H), 2.09 (s, 3H);

^13^C NMR (101 MHz, DMSO-*d_6_*) δ 169.1, 164.2, 154.7, 148.8, 141.7, 136.3, 130.6, 129.2, 129.1, 128.7, 128.5, 127.2, 126.3, 124.3, 120.7, 119.4, 111.1, 24.6;

MS: ESI +ve = 370 (M+Na);

Micro analysis – calculated for C_20_H_17_N_3_O_3_: C 69.15, H 4.93, N 12.10; found: C 69.45; H 4.98; N 11.69.

***(E)-3-hydroxy-N'-((2-methyl-1H-indol-3-yl)methylene)-2-naphthohydrazide (11a)***

Starting from 3-hydroxy-2-naphthoic hydrazide (0.202 g, 1 mmol) and 2-methylindole-3-carboxaldehyde (0.159 g, 1 mmol), **11a** (0.111 g, 32%) was obtained as a pale yellow solid (m.p. 219–220 °C);

^1^H NMR (400 MHz, DMSO-*d_6_*) δ 11.80 (bs, 2H), 11.56 (s, 1H), 8.76 (s, 1H), 8.56 (s, 1H), 8.32 – 8.25 (m, 1H), 7.92 (d, *J* = 8.2 Hz, 1H), 7.78 (d, *J* = 8.2 Hz, 1H), 7.52 (t, *J* = 7.5 Hz, 1H), 7.41 – 7.32 (m, 3H), 7.22 – 7.11 (m, 2H), 2.57 (s, 3H);

^13^C NMR (101 MHz, DMSO-*d_6_*) δ 164.1, 155.6, 146.1, 141.2, 136.4, 136.2, 129.8, 129.1, 128.64, 127.2, 126.3, 125.9, 124.2, 122.4, 121.7, 120.9, 119.7, 111.4, 111.2, 107.9, 11.99;

MS: ESI +ve = 344 (M+1);

Micro analysis – calculated for C_21_H_17_N_3_O_2_: C 73.45, H 4.99, N 12.24; found: C 73.02; H 5.12; N 12.77.

***3-Hydroxynaphthalene-2-carboxylic acid quinoxaline-2-ylmethylenehydrazide*** ***(12a)***

Starting from 3-hydroxy-2-naphthoic hydrazide (0.202 g, 1 mmol) and quinoxaline-2-carbaldehyde (0.158 g, 1 mmol), **12a** (0.183 g, 53%) was obtained as a pale yellow solid (m.p. 299–300 °C);

^1^H NMR (400 MHz, DMSO-*d_6_*) δ ^1^H NMR (DMSO-*d_6_*): *δ* 11.84 (bs, 1H), 9.50 (s, 1H); 8.63 (s, 1H), 8.43 (s, 1H), 8.26 (s, 1H), 8.12 (s, 1H), 7.91 (d, *J* = 8.3 Hz, 2H), 7.75 (dd, *J* = 8.3, 8.3 Hz, 2H), 7.48 (dd, *J* = 7.4, 7.4 Hz, 2H), 7.35 (d, *J* = 7.6 Hz, 2H), 7.29 (s, 1H);

^13^C NMR (101 MHz, DMSO-*d_6_*) δ 160.3, 158.3, 153.6, 151.7, 148.4, 142.8, 141.2, 135.8, 130.7, 130.4, 128.9, 128.5, 128.1, 126.7, 125.8, 123.7, 121.2, 110.4, 105.8;

MS: ESI +ve = 342 (M);

Micro analysis – calculated for C_20_H_14_N_4_O_2_: C 70.17, H 4.12, N 16.37; found: C 70.42; H 4.32; N 16.17.

***(E)-N'-(benzo[c][1,2,5]oxadiazol-5-ylmethylene)-3-hydroxy-2-naphthohydrazide (13a)***

Starting from 3-hydroxy-2-naphthoic hydrazide (0.202 g, 1 mmol) and 2,1,3-benzoxadiazole-5-carbaldehyde (0.148 g, 1 mmol), **13a** (0.172 g, 51%) was obtained as a yellow solid (m.p. 292–293 °C);

^1^H NMR (400 MHz, DMSO-*d_6_*) δ 12.17 (bs, 1H), 11.24 (bs, 1H), 8.61 (s, 1H), 8.46 (s, 1H), 8.31 (s, 1H), 8.18 (d, *J* = 9.7 Hz, 1H), 8.13 (d, *J* = 9.5 Hz, 1H), 7.94 (d, *J* = 8.2 Hz, 1H), 7.78 (d, *J* = 8.3 Hz, 1H), 7.53 (t, *J* = 7.5 Hz, 1H), 7.38 (d, *J* = 7.5 Hz, 1H), 7.35 (s, 1H).

^13^C NMR (101 MHz, DMSO-*d_6_*) δ 164.4, 154.2, 149.7, 149.6, 146.4, 139.0, 136.4, 131.1, 130.0, 129.2, 128.8, 127.3, 126.3, 124.3, 121.2, 117.2, 117.2, 111.0;

MS: ESI +ve = 324 (M+1);

Micro analysis – calculated for C_18_H_12_N_4_O_3_: C 65.06, H 3.64, N 16.86; found C 65.32, H 3.86, N 16.67.

***(E)-N'-(benzo[c][1,2,5]thiadiazol-5-ylmethylene)-3-hydroxy-2-naphthohydrazide (14a)***

Starting from 3-hydroxy-2-naphthoic hydrazide (0.202 g, 1 mmol) and benzo[*c*][1,2,5]thiadiazole-5-carbaldehyde (0.164 g, 1 mmol), **13a** (0.162 g, 46%) was obtained as a pale yellow solid (m.p. 290–292 °C);

^1^H NMR (400 MHz, DMSO-*d_6_*) δ 12.07 (bs, 1H), 11.46 (bs, 1H), 8.67 (s, 1H), 8.47 (s, 1H), 8.35 (s, 1H), 8.30 (dd, *J* = 9.3, 1.0 Hz, 1H), 8.17 (d, *J* = 9.2 Hz, 1H), 7.94 (d, *J* = 8.2 Hz, 1H), 7.78 (d, *J* = 8.3 Hz, 1H), 7.52 (t, *J* = 7.4 Hz, 1H), 7.40 – 7.33 (m, 2H);

^13^C NMR (101 MHz, DMSO-*d_6_*) δ 164.4, 154.0, 148.9, 146.7, 143.4, 142.1, 141.8, 136.3, 131.4, 131.1, 129.5, 129.1, 128.7, 127.3, 126.3, 124.3, 121.8, 111.0;

MS: ESI −ve = 324 (M−1);

Micro analysis - calculated for C_18_H_12_N_4_O_2_S: C 62.06, H 3.47, N 16.08; found C 62.32, H 3.72, N 16.17.
