# Supplementary figures and images for: Building a Better Dynasore: The Dyngo Compounds Potently Inhibit Dynamin and Endocytosis
Source: Traffic. 2013 Oct 9;14(12):1272–89. doi: 10.1111/tra.12119 (PMC4138991; doi:10.1111/tra.12119)

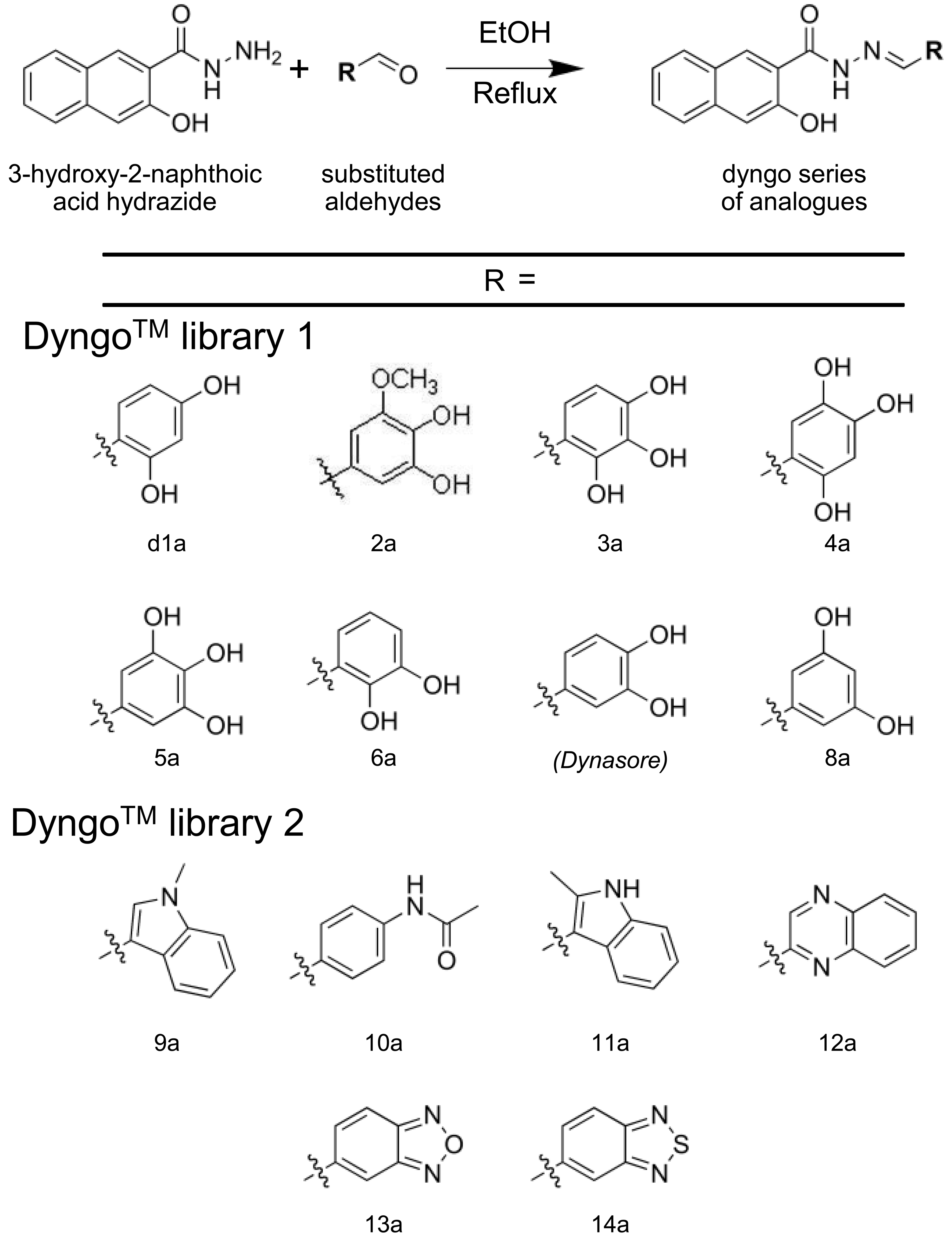
**Scheme S1.** *Scheme for the synthesis of Dyngo analogues.*

Supplement: Supplementary file 3 — Scheme S1. Scheme for the synthesis of Dyngo analogs. [file tra-14-1272-s3.docx]
