# Supplementary material for: Building a Better Dynasore: The Dyngo Compounds Potently Inhibit Dynamin and Endocytosis
Source: Traffic. 2013 Oct 9;14(12):1272–89. doi: 10.1111/tra.12119 (PMC4138991; doi:10.1111/tra.12119)
Supplement: Supplementary file 4 — Figure S1. Dynasore is a poor dynamin I inhibitor when assayed in the presence of Tween‐80. A) Structure of dynasore. B) Dose‐dependent inhibition by dynasore of dynamin I GTPase activity stimulated by PS liposomes in the presence of Tween‐80. C) IC50 values of dynamin I after activation by four mechanisms in the presence of Tween‐80. Dynasore was either produced in house (synthesized), purchased from Sigma or obtained from the laboratory of Tom Kirchhausen (TK). Dynasore was tested at a range of concentrations up to a maximum of 1 mM, with the exception of data marked with *, which were tested up to 1.5 mM. D) Effect of dynasore on endocytosis of Tfn‐A594 in U2OS cells. All data are means ± SEM of three independent experiments. [file tra-14-1272-s4.docx]

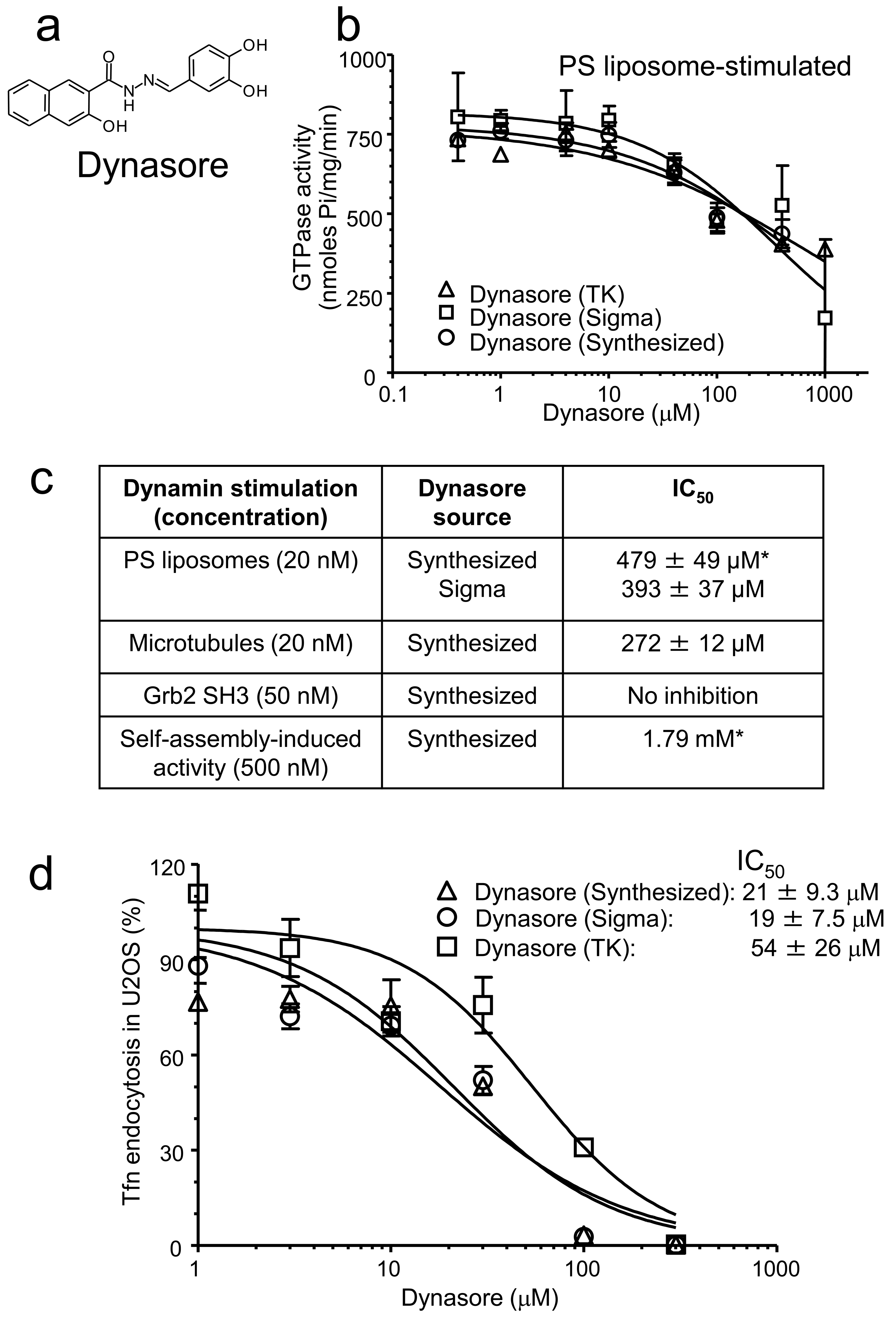
**Figure S1.** *Dynasore is a poor dynamin I inhibitor when assayed in the presence of Tween-80*. (a) Structure of dynasore. (b) Dose-dependent inhibition by dynasore of dynamin I GTPase activity stimulated by PS liposomes in the presence of Tween-80. (c) IC_50_ values of dynamin I after activation by four mechanisms in the presence of Tween-80. Dynasore was either produced in house (‘synthesized’), purchased from Sigma or obtained from the laboratory of Tom Kirchhausen (TK). Dynasore was tested at a range of concentrations up to a maximum of 1 mM, with the exception of data marked with * which were tested up to 1.5 mM. (d) Effect of dynasore on endocytosis of Tfn-A594 in U2OS cells. All data are means ± S.E.M. of 3 independent experiments.
