# Supplementary material for: Building a Better Dynasore: The Dyngo Compounds Potently Inhibit Dynamin and Endocytosis
Source: Traffic. 2013 Oct 9;14(12):1272–89. doi: 10.1111/tra.12119 (PMC4138991; doi:10.1111/tra.12119)
Supplement: Supplementary file 5 — Figure S2. Dyngo compound 4a has no effect on dynamin binding to SH3 domains. Pull down of dynamin I in the absence or presence of the indicated 4a concentrations was performed using the SH3 domains of Grb2, endophilin I or amphiphysin I attached to GSH beads. The proteins were resolved on 12% SDS‐PAGE gels and visualized using Coomasie Blue. The results are shown for one experiment performed in triplicate and the same results were obtained in two further independent experiments (in duplicate). [file tra-14-1272-s5.docx]

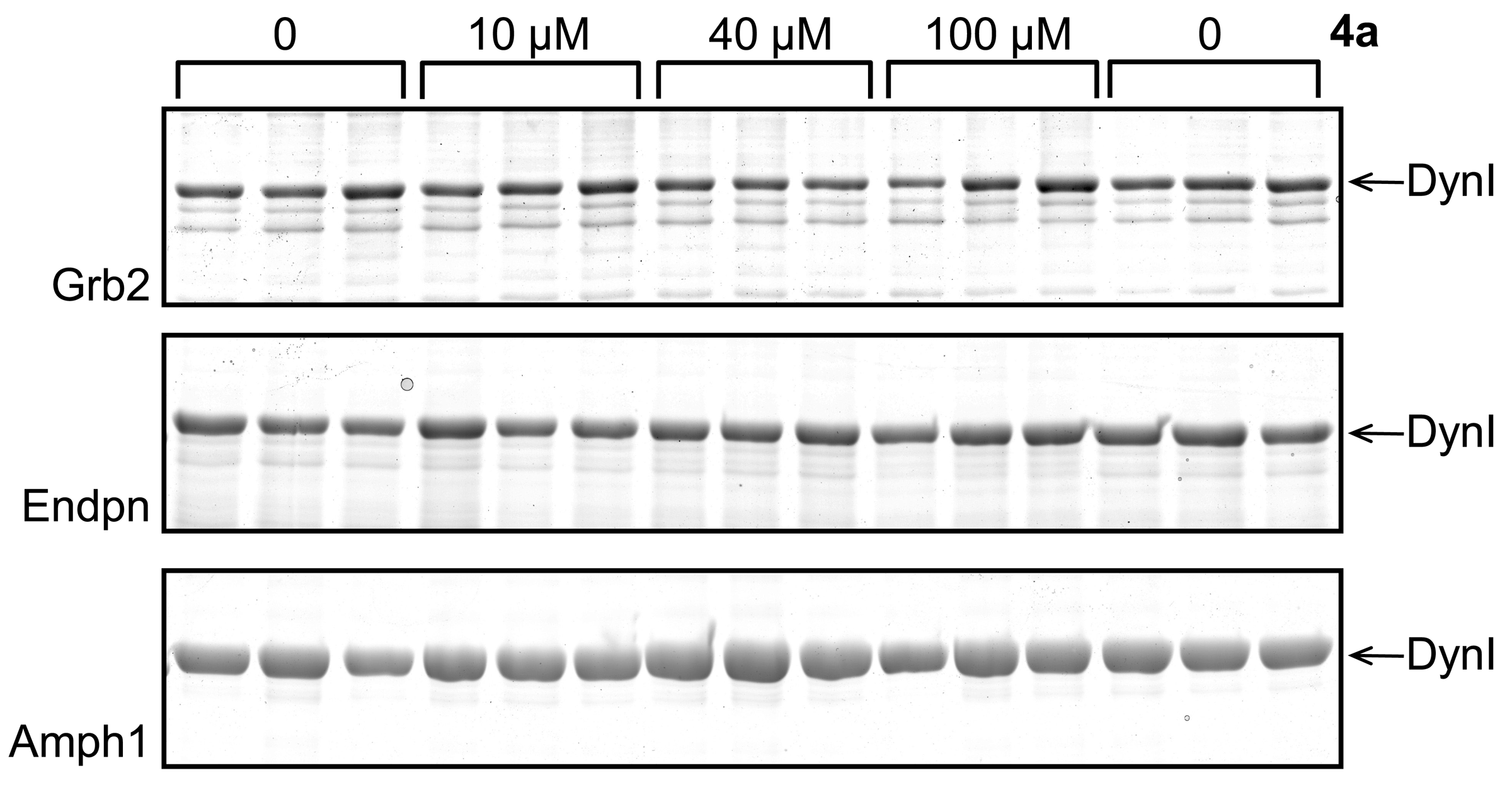
**Figure S2.** – *Dyngo compound* ***4a*** *has no effect on dynamin binding to SH3 domains.*  Pull down of dynamin I in the absence of presence of the indicated **4a** concentrations was performed using the SH3 domains of Grb2, Endophilin I or Amphiphysin I attached to GSH beads. The proteins were resolved on 12% SDS-PAGE gels and visualised using Coomasie Blue. The results are shown for 1 experiment performed in triplicate and the same results were obtained in two further independent experiments (in duplicate).
