# Supplementary material for: Building a Better Dynasore: The Dyngo Compounds Potently Inhibit Dynamin and Endocytosis
Source: Traffic. 2013 Oct 9;14(12):1272–89. doi: 10.1111/tra.12119 (PMC4138991; doi:10.1111/tra.12119)
Supplement: Supplementary file 6 — Figure S3. Dyngo compounds do not affect amphiphysin protein–protein interactions. The effect of dynasore and Dyngo compounds on binding of clathrin heavy‐chain C‐terminal domain or AP‐2 alpha ear domain to amphiphysin 1 PRD + CLAP domains determined by ELISA assays. Data are mean and error bars represent SEM for triplicate measurements for n = 1. [file tra-14-1272-s6.docx]

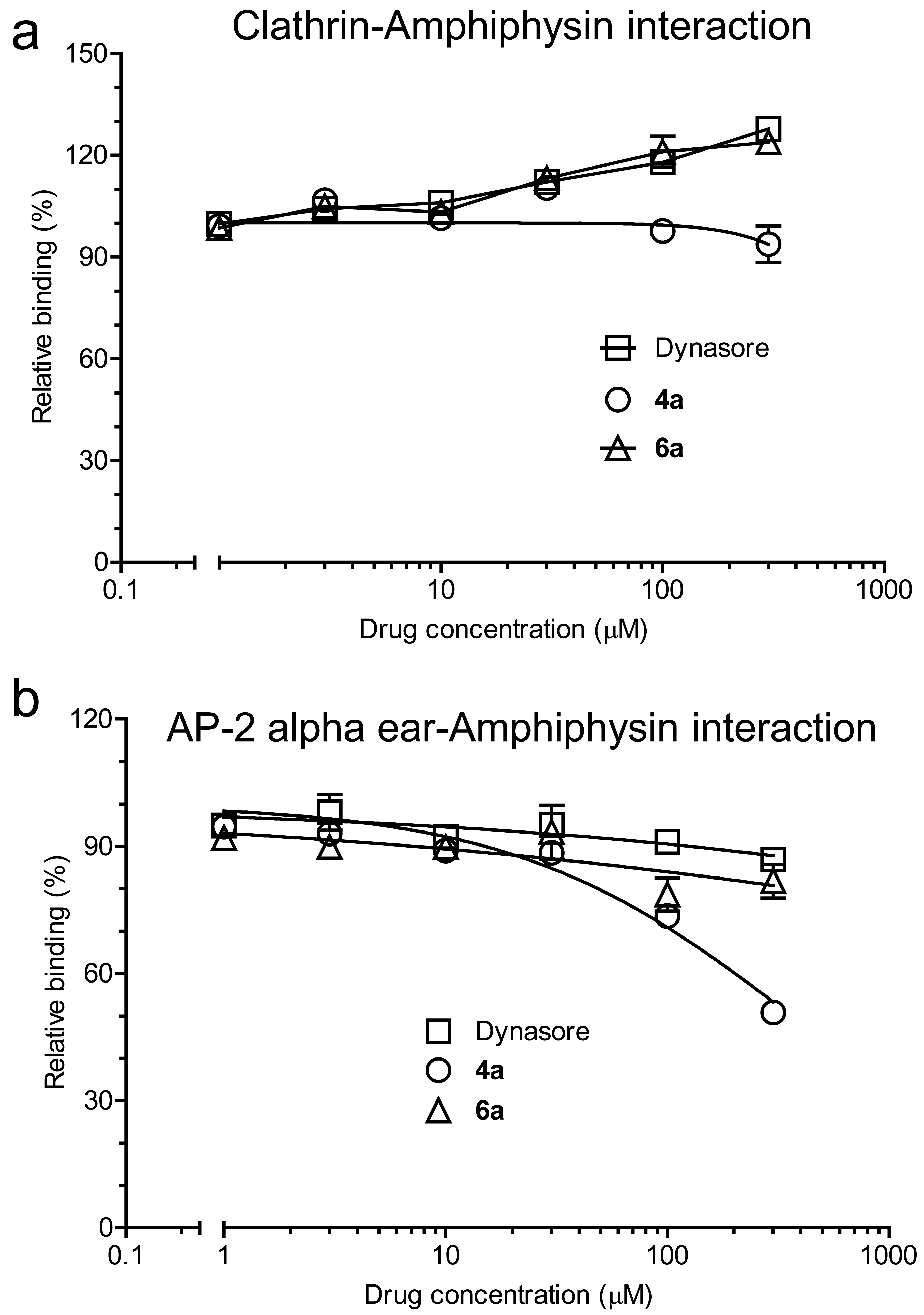
**Figure S3.** *Dyngo compounds do not affect amphiphysin protein-protein interactions.* The effect of dynasore and *Dyngo* compound*s* on binding of clathrin heavy chain C-terminal domain or AP-2 alpha ear domain to amphiphysin 1 PRD+CLAP domains determined by ELISA assays. Data are mean and error bars represent SEM for triplicate measurements for n-=1.
