# Supplementary material for: Building a Better Dynasore: The Dyngo Compounds Potently Inhibit Dynamin and Endocytosis
Source: Traffic. 2013 Oct 9;14(12):1272–89. doi: 10.1111/tra.12119 (PMC4138991; doi:10.1111/tra.12119)
Supplement: Supplementary file 7 — Figure S4. Dyngo series 4a, 6a and dynasore are non‐toxic and do not affect cell viability in HeLa cells. A and B) HeLa cells were exposed to MiTMAB or the indicated Dyngo compound for 8 h in the presence (A) and absence of serum (B) and then analyzed using an LDH assay. Data represent SEM (n = 2 independent experiments). C–F) Cell membrane integrity as an indicator of viability (C and E) and cell proliferation (D and F) in HeLa cells were analyzed after prolonged exposure (20 h) to 4a, 6a and dynasore in the presence (C and D) and absence of serum (E and F) using a trypan blue exclusion assay. Data represent SEM (n = 2 independent experiments). [file tra-14-1272-s7.docx]

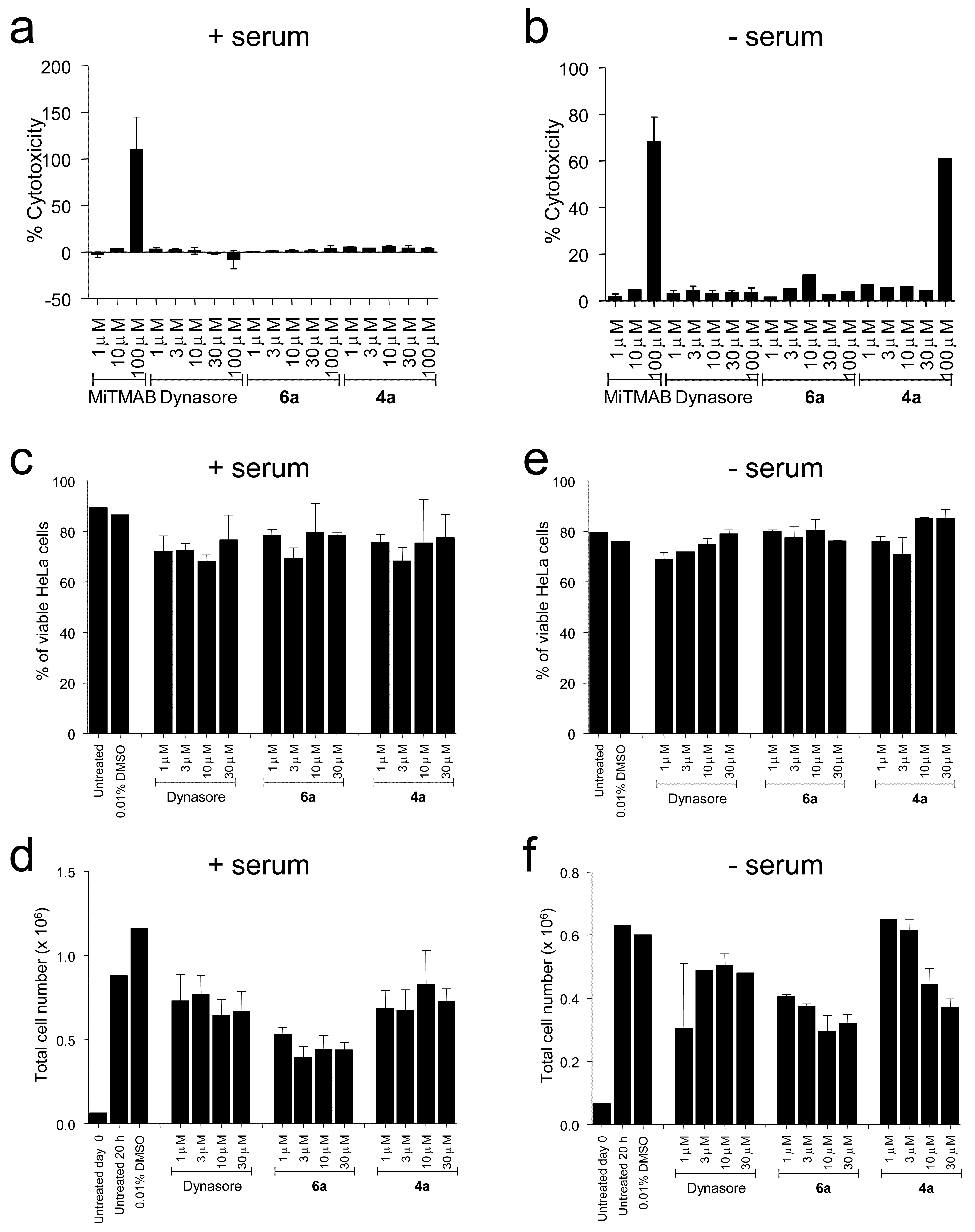
**Figure S4.** *Dyngo* series **4a**, -**6a** and dynasore are non-toxic and do not affect cell viability in HeLa cells. (a-b) HeLa cells were exposed to MiTMAB or the indicated Dyngo compound for 8 h in the presence (a) and absence of serum (b) then analyzed using an LDH assay. Data represent SEM (n=2 independent experiments). (c-f) Cell membrane integrity as an indicator of viability (c and e) and cell proliferation (d and f) in HeLa cells were analyzed after prolonged exposure (20 h) to **4a**, -**6a** and dynasore in the presence (c and d) and absence of serum (e and f) using a trypan blue exclusion assay. Data represent SEM (n=2 independent experiments).
