# Supplementary material for: Building a Better Dynasore: The Dyngo Compounds Potently Inhibit Dynamin and Endocytosis
Source: Traffic. 2013 Oct 9;14(12):1272–89. doi: 10.1111/tra.12119 (PMC4138991; doi:10.1111/tra.12119)
Supplement: Supplementary file 8 — Figure S5. Effect of dynasore analogs on mitochondria in HeLa cells. A) HeLa cells stably expressing H2B‐mCherry (red) were serum‐starved, incubated with Mitotracker Green FM (green) and imaged by confocal microscopy. The left panel shows cells at 40× magnification, while the right panel shows greater detail of mitochondrial structure. All nuclei exhibited red fluorescence, although the intensity varied considerably. Cells were then treated with either DMSO (B), 30 μM 4a (C), 100 μM dynasore (D) or 30 μM 6a. In (B) to (E), left‐hand panels show images acquired 30 min after treatment, central panels show a more detailed image of mitochondria after 30 min of treatment and the right‐hand panels show the cells after 60 min. After 30 min of treatment, 4a‐ and dynasore‐treated cells exhibited unchanged mitochondrial morphology, including elongated mitochondria (arrows in A–D), while 6a‐treated cells exhibited relatively fragmented mitochondria (arrows in E). After 60 min of treatment, all treated cells exhibited a reduction in Mitotracker Green FM fluorescence. Scale bars = 20 µm for images in left‐ and right‐hand panels, while for zoomed panels the scale bar = 5 µm. [file tra-14-1272-s8.docx]

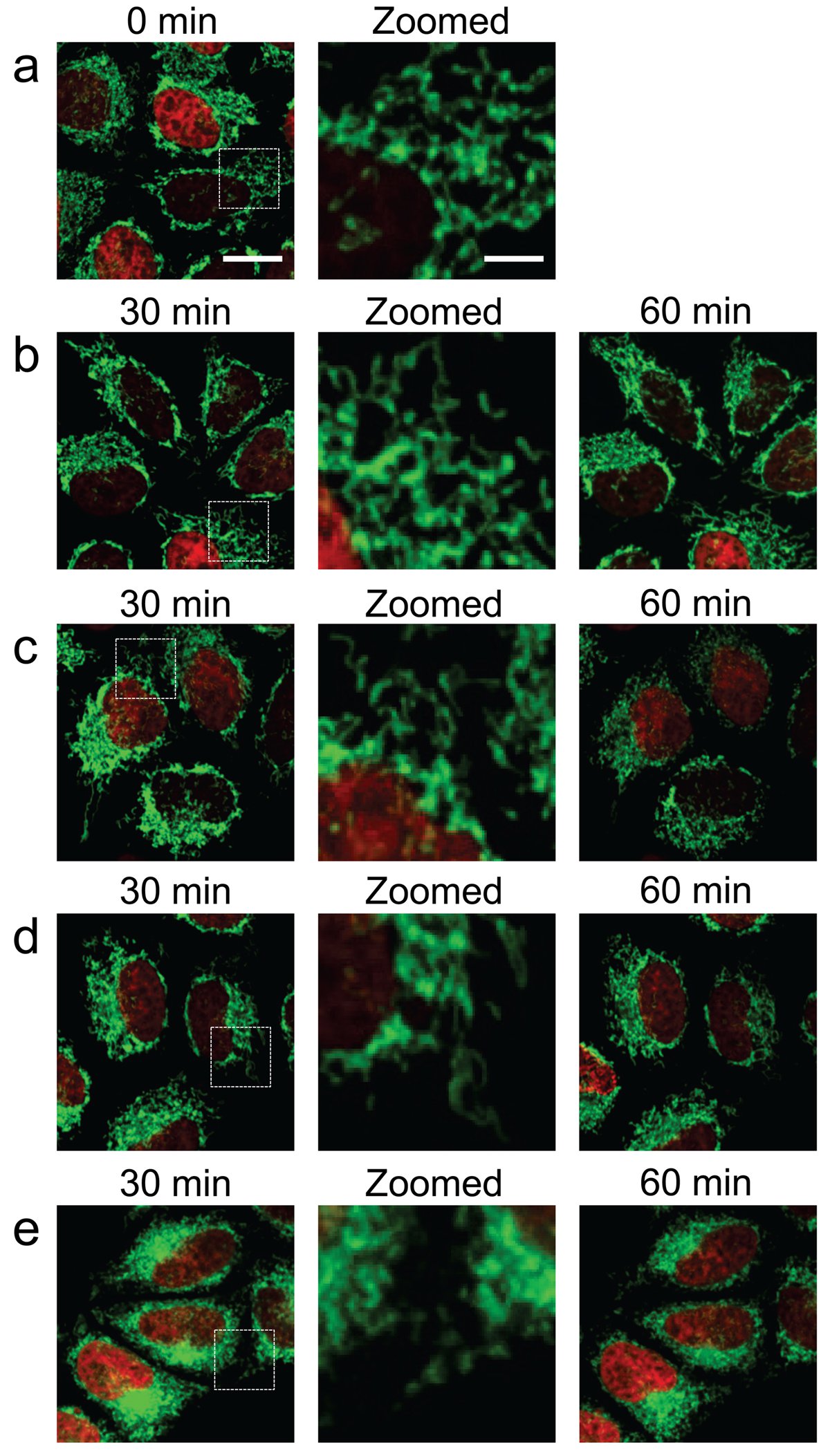
**Figure S5.** Effect of dynasore analogues on mitochondria in HeLa cells. (a) HeLa cells stably expressing H2B-mCherry (red) were serum-starved, incubated with Mitotracker Green FM (green) and imaged by confocal microscopy. The left panel shows cells at 40 x magnification, while the right panel shows greater detail of mitochondrial structure. All nuclei exhibited red fluorescence, although the intensity varied considerably. Cells were then treated with either DMSO (b), 30 μM **4a** (c), 100 μM dynasore (d) or 30 μM **6a**. In (b) to (e), left hand panels show images acquired 30 min after treatment, central panels show a more detailed image of mitochondria after 30 min of treatment, and the right hand panels show the cells after 60 min. After 30 min of treatment, **4a**- and dynasore-treated cells exhibited unchanged mitochondrial morphology, including elongated mitochondria (arrows in a to d) while **6a**-treated cells exhibited relatively fragmented mitochondria (arrows in e). After 60 min of treatment, all treated cells exhibited a reduction in Mitotracker Green FM fluorescence. Scale bars = 20 μm for images in left and right hand panels, while for zoomed panels the scale bar = 5 μm.
