# Supplementary material for: Building a Better Dynasore: The Dyngo Compounds Potently Inhibit Dynamin and Endocytosis
Source: Traffic. 2013 Oct 9;14(12):1272–89. doi: 10.1111/tra.12119 (PMC4138991; doi:10.1111/tra.12119)
Supplement: Supplementary file 9 — Figure S6. U2OS cells express only dynamin II. Equal protein load (50 µg) from four different cancer cell lines was run on SDS gels along with 0.2 µg partially purified full‐length recombinant dynamin I, II or III. The three dynamins were detected with isoform‐specific antibodies by western blot. Results shown are for one experiment with duplicate or triplicate cell samples and similar results were obtained in two additional experiments. [file tra-14-1272-s9.docx]

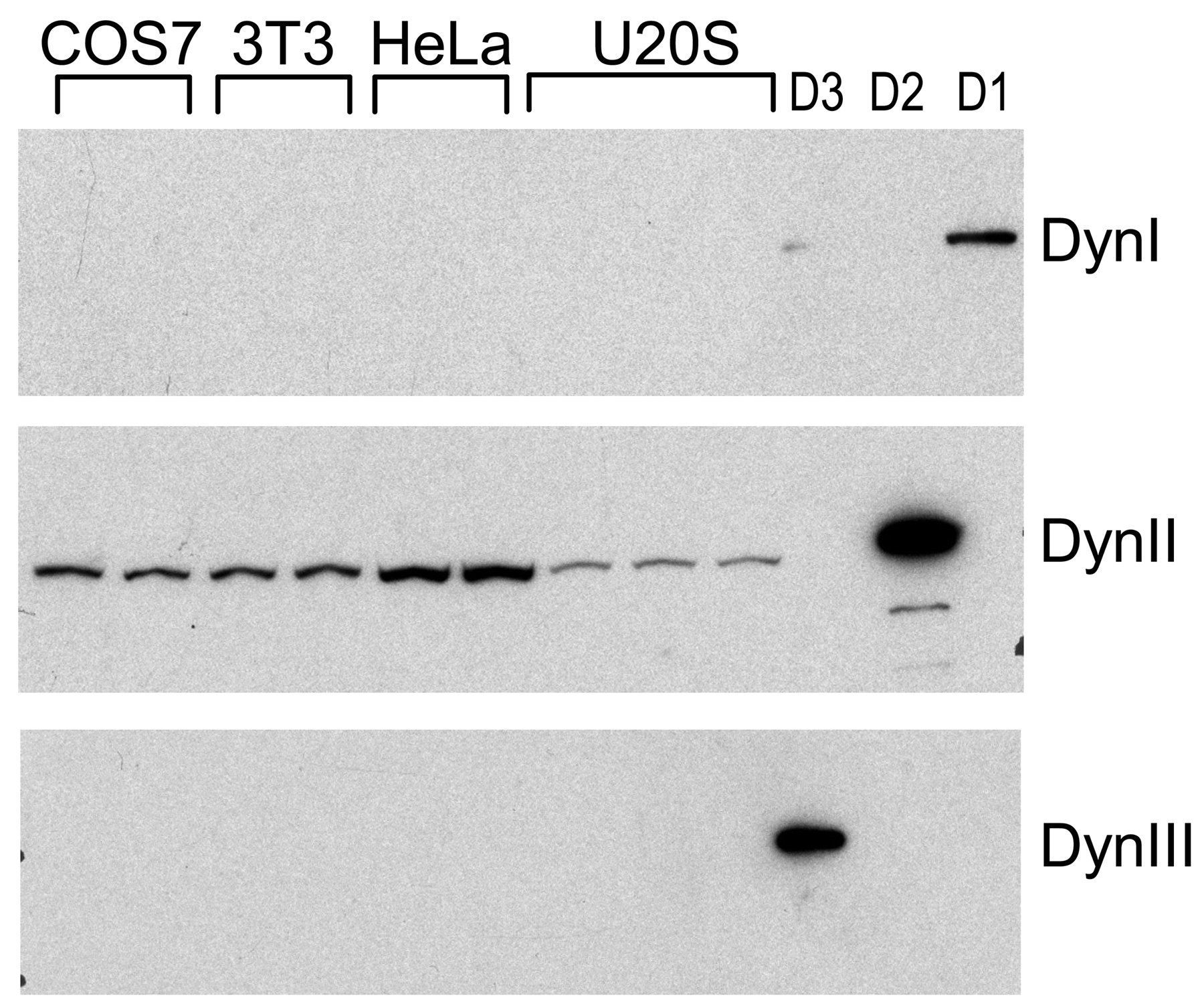


**Figure S6.** *U2OS cells express only dynamin II.* Equal protein load (50 µg) from 4 different cancer cell lines were run on SDS gels along with 0.2 µg partially purified full-length recombinant dynamin I, II or III. The three dynamins were detected with isoform-specific antibodies by Western blot. Results shown are for 1 experiment with duplicate or triplicate cell samples and similar results were obtained in two additional experiments.
