# Supplementary material for: Building a Better Dynasore: The Dyngo Compounds Potently Inhibit Dynamin and Endocytosis
Source: Traffic. 2013 Oct 9;14(12):1272–89. doi: 10.1111/tra.12119 (PMC4138991; doi:10.1111/tra.12119)
Supplement: Supplementary file 10 — Figure S7. Dyngo compound 4a does not block dynamin‐independent endocytosis of cholera toxin. A) NIH3T3 cells were serum starved for 3 h in unsupplemented DMEM. Cells were subsequently pretreated (or not) for 20 min with 20, 50 or 80 μM 4a or dynasore. Cells were next incubated with 5 µg/mL Tfn‐488 and 2 µg/mL CT‐555 in the continued presence of 20, 50 or 80 μM 4a or dynasore for 5 min at 37°C. 2 × 1‐min washes with 0.5 M glycine and pH 2.2 were performed to remove surface labeling of CT‐555 prior to fixation in 4% paraformaldehyde. Cells were imaged on a 510 Meta Zeiss confocal microscope. Scale bar is 10 µm. B) Over 50 cells treated with each condition in (A) were imaged and fluorescence intensity was calculated based on each unique histogram profile. Each treated sample was calculated as a percentage of control units. All data are means ± SEM. [file tra-14-1272-s10.docx]

**
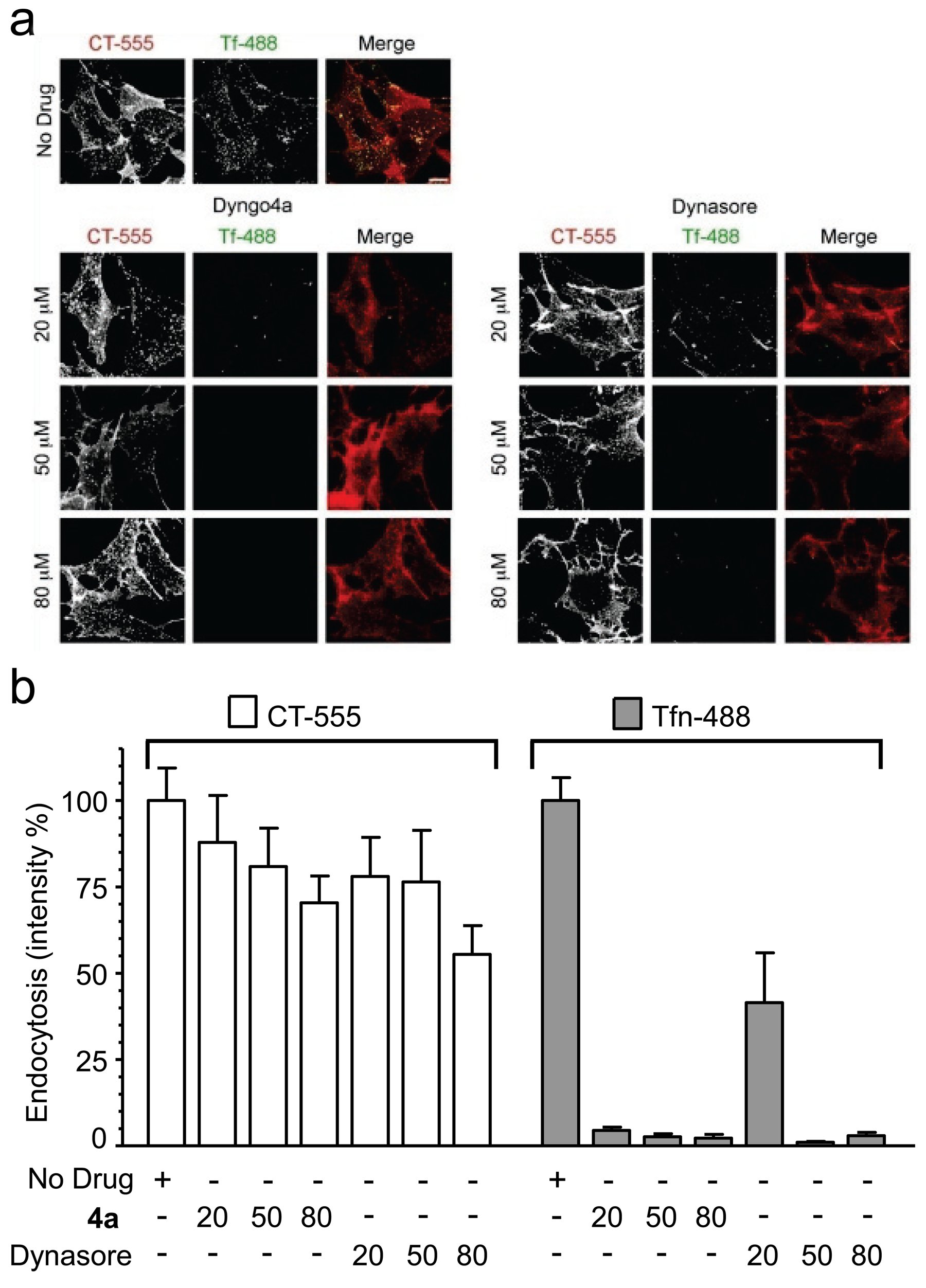
**

**Figure S7.** *Dyngo compound* ***4a*** *does not block dynamin-independent endocytosis of cholera toxin.* (a) NIH3T3 cells were serum starved for 3 hours in unsupplemented DMEM. Cells were subsequently pre-treated (or not) for 20 min with 20, 50 or 80 μM **4a** or dynasore. Cells were next incubated with 5 μg/ml Tfn-488 and 2 μg/ml CT-555 in the continued presence of 20, 50 or 80 μM **4a** or dynasore for 5 min at 37 °C. 2 × 1 min washes with 0.5 M glycine, pH 2.2 were performed to remove surface labelling of CT-555 prior to fixation in 4% paraformaldehyde. Cells were imaged on a 510 Meta Zeiss confocal microscope. Scale bar is 10 μm. (b) Over 50 cells treated with each condition in (a) were imaged and fluorescence intensity was calculated based on each unique histogram profile. Each treated sample was calculated as a percentage of control units. All data are means ± S.E.M.
