# Supplementary material for: Building a Better Dynasore: The Dyngo Compounds Potently Inhibit Dynamin and Endocytosis
Source: Traffic. 2013 Oct 9;14(12):1272–89. doi: 10.1111/tra.12119 (PMC4138991; doi:10.1111/tra.12119)
Supplement: Supplementary file 11 — Figure S8. Blockade of synaptic vesicle turnover in CGNs. A–D) Activity‐dependent loading and unloading of the styryl dye FM1‐43 in CGN cultures. Representative images either loaded at S1 (A) or S2 (B) in the absence of 4a or in its presence (S1—panel C; S2—panel D) are displayed. Scale bar represents 1 µm. E and F) Dextran endocytosis specifically reflects ADBE relative to CME. Representative images of control (E) or 4a (panel F, 30 μM, 15‐min preincubation) inhibition of uptake of fluorescent tetramethyrhodamine‐dextran (50 μM) in CGNs electrically stimulated by a train of 800 action potentials (80 Hz) followed by immediate dextran washout. [file tra-14-1272-s11.docx]

**
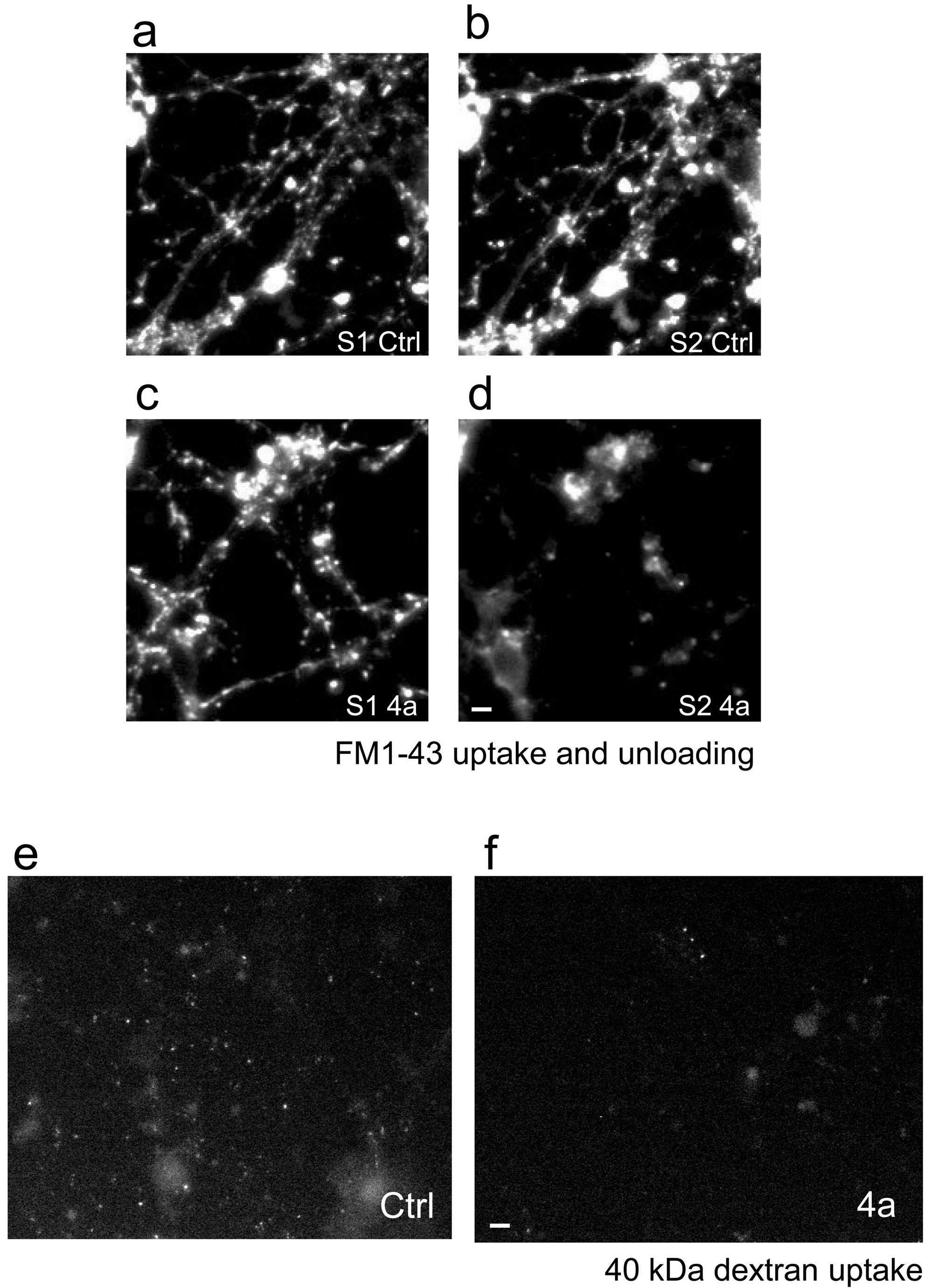
**

**Figure S8.** *Blockade of synaptic vesicle turnover in CGNs. a-d)* Activity-dependent loading and unloading of the styryl dye FM1-43 in CGN cultures. Representative images are displayed of either loaded at S1 (a) or S2 (b) in the absence of **4a**, or in its presence (S1 – panel c; S2 – panel d). Scale bar represents 1 μm. e-f) Dextran endocytosis specifically reflects ADBE relative to CME. Representative images of Control (e) or **4a** (panel f, 30 μM, 15 min preincubation) inhibition of uptake of fluorescent tetramethyrhodamine-dextran (50 µM) in CGNs electrically stimulated by a train of 800 action potentials (80 Hz) followed by immediate dextran washout.
