# Supplementary material for: Building a Better Dynasore: The Dyngo Compounds Potently Inhibit Dynamin and Endocytosis
Source: Traffic. 2013 Oct 9;14(12):1272–89. doi: 10.1111/tra.12119 (PMC4138991; doi:10.1111/tra.12119)
Supplement: Supplementary file 12 — Figure S9. Dynasore inhibits SVE in neurons. The effect of dynasore on whole‐cell membrane capacitance was investigated at the Calyx of Held in parallel with the experiments in Figure 4 except that there was 0.8 mM dynasore in the puffing pipette. A) A sample trace shows membrane capacitance on control (black) and dynasore‐treated samples (red). B) Collated data of normalized capacitance measurement from control and dynasore‐treated neurons (n = 7). [file tra-14-1272-s12.docx]

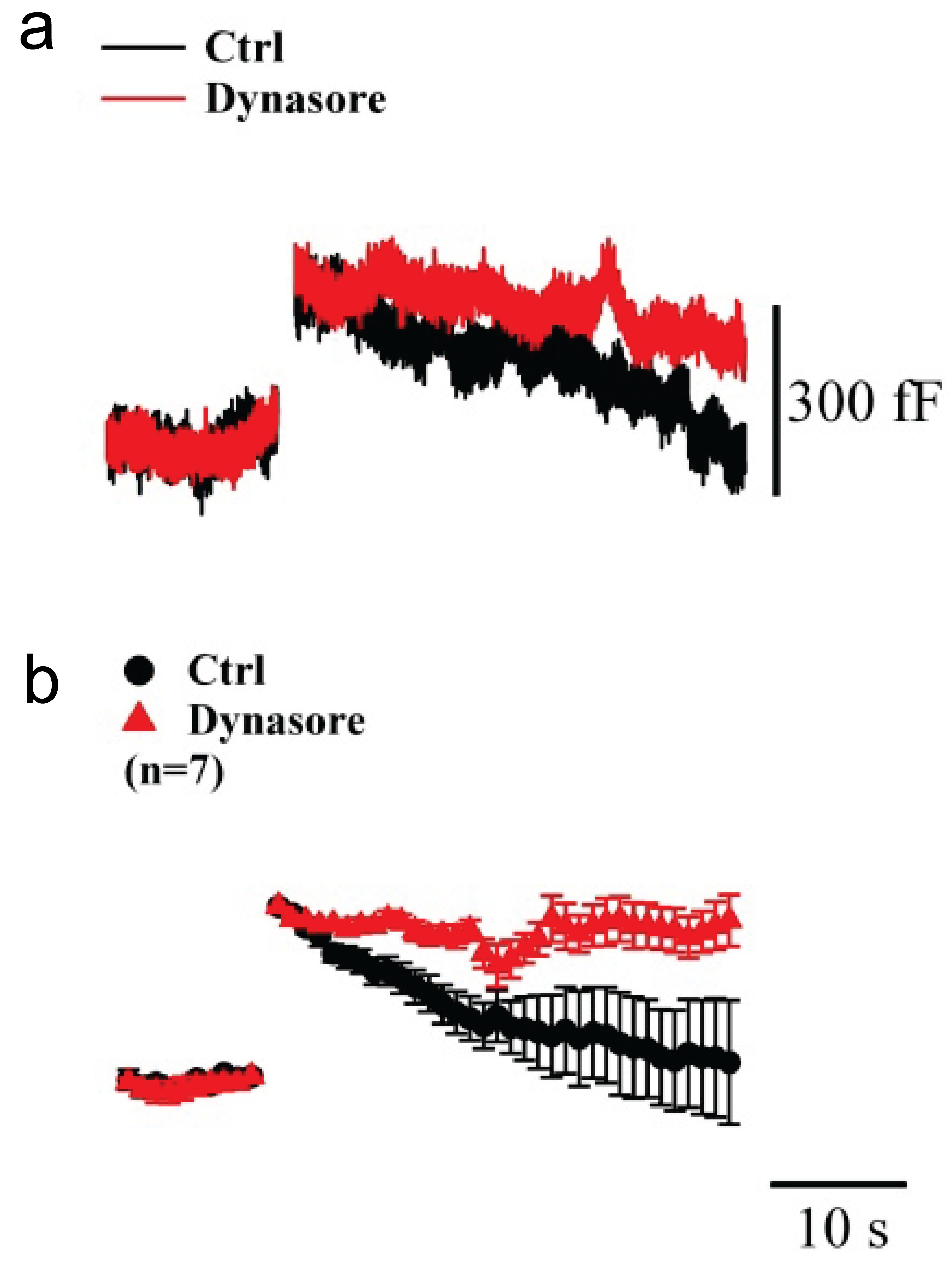


**Figure S9.** *Dynasore inhibits SVE in neurons.* The effect of dynasore on whole cell membrane capacitance was investigated at the Calyx of Held in parallel with the experiments in Fig 4 except that there was 0.8 mM dynasore in the puffing pipette. (a) A sample trace shows membrane capacitance on control (black) and dynasore treated samples (red). (b) Collated data of normalized capacitance measurement from control and dynasore treated neurons (n=7).
